# Supplementary material for: BIN1 recovers tauopathy-induced long-term memory deficits in mice and interacts with Tau through Thr348 phosphorylation
Source: Acta Neuropathol. 2019 May 7;138(4):631–52. doi: 10.1007/s00401-019-02017-9 (PMC6778065; doi:10.1007/s00401-019-02017-9)
Supplement: Supplementary file 1 — Supplementary material 1 (DOCX 12215 kb) [file 401_2019_2017_MOESM1_ESM.docx]

**BIN1 recovers tauopathy-induced long-term memory deficits in mice and interacts with Tau through Thr^348^ phosphorylation**

Maxime Sartori^1,2,3,4,^*, Tiago Mendes^5,6,7,8,^*, Shruti Desai^5,6,7,^*, Alessia Lasorsa^7,9^, Adrien Herledan^6,10,11^, Nicolas Malmanche^5,6,7^, Petra Mäkinen^12^, Mikael Marttinen^12^, Idir Malki^7,9^, Julien Chapuis^5,6,7^, Amandine Flaig^5,6,7^, Anaïs-Camille Vreulx^5,6,7^, Marion Ciancia^1,2,3,4^, Philippe Amouyel^5,6,7^, Florence Leroux^6,10,11^, Benoit Déprez^6,10,11^, François-Xavier Cantrelle^7,9^, Damien Maréchal^1,2,3,4^, Laurent Pradier^8^, Mikko Hiltunen^12^, Isabelle Landrieu^7,9^, Devrim Kilinc^5,6,7,#^, Yann Herault^1,2,3,4,#^, Jocelyn Laporte^1,2,3,4,#^, Jean-Charles Lambert^5,6,7,#^

Supplementary Information

* Maxime Sartori, Tiago Mendes, and Shruti Desai contributed equally to this work.

# Devrim Kilinc, Yann Herault, Jocelyn Laporte and Jean-Charles Lambert contributed equally to this work.

^1^ Institut de Génétique et de Biologie Moléculaire et Cellulaire (IGBMC), Illkirch, France

^2^ INSERM U1258, Illkirch, France

^3^ CNRS UMR7104, Illkirch, France

^4^ Strasbourg University, Illkirch, France

^5^ INSERM, U1167, RID-AGE-Risk Factors and Molecular Determinants of Aging-Related Diseases, Lille, France

^6^ Institut Pasteur de Lille, Lille, France

^7^ University of Lille, DISTALZ Laboratory of Excellence (LabEx), Lille, France

^8^ SANOFI Neurosciences, Chilly-Mazarin, France

^9^ CNRS UMR8576, Lille, France

^10^ University of Lille, EGID, Lille, France

^11^ Inserm, U1177, Lille, France

^12^ Institute of Biomedicine, University of Eastern Finland, Kuopio, Finland

**Supplementary Materials and Methods**

**HCS image segmentation in Columbus**

HCS was performed in the IN Cell platform. IN Cell image registration and transfer files (.xdce files) were manually edited to import images to Columbus only from control wells, thereby generating the so-called “control plates” for script optimization and plate validation. HCS image analysis was performed using Columbus software (Fig. S5). In order to improve the detection of Tau and MAP2 areas, somatic regions were excluded from the Tau (Cy5 channel) and MAP2 (FITC channel) images by removing the Hoechst signal (DAPI channel), using the formula $A\times\left( if\left( B_{median}+0.1\times B_{stddev} \right),1,0 \right)$, where *A* is the Cy5 or the FITC channel and *B* is the DAPI channel. Tau and MAP2 areas were calculated after thresholding using the formula $(if(A>\left( A_{mean}-q_{1}\times A_{stddev}),1,0 \right))$, where *A* is the somata-excluded Cy5 or FITC channels and *q_1_* is area threshold coefficient in terms of SD. For the FITC channel (MAP2), the parameter *q_1_* had a constant value of 0.1. For the Cy5 channel (Tau), *q_1_* was one of the optimization parameters determined through an iterative process, *i.e.*, script optimization. PLA spots (dsRed channel) in the thresholded Tau area using the *Find Spots* and *Select Population* building blocks of Columbus following *Method D*. Here, the *Splitting Coefficient* was kept constant at 0.9, *Spot Area* was restrained between 15 and 60 px^2^, and the upper threshold of *Spot Contrast* was kept constant at 0.9. In addition, three parameters of this building block were determined through script optimization: q_2_, threshold of *Detection Sensitivity*; q_3_, threshold of *Background Correction*; and q_4_, lower threshold of *Spot Contrast*.

**Iterative process to determine optimum parameters for image segmentation script**

Each control plate was used to optimize the image segmentation parameters to be used to analyze the corresponding full plate. Multiple Columbus analysis scripts were created by assigning distinct values combinatorially to each of the optimization parameter (q_1_-q_4_). For example, assigning three distinct values per parameter results in 3^4^ = 81 combinations; hence the optimization was performed by running Columbus in the *Batch Analysis* mode, using 81 analysis scripts. This resulted in 81 result files per control plate, which were then analyzed in MATLAB. Data obtained for each well of the control plate were corrected for spatial bias (horizontal), using the slope of the line that fits the column averages based on the least-squares method. For each control plate, three values typically used in HCS analysis (Bray and Carpenter, 2013) were calculated: (i) strictly standardized mean difference, β factor, $\beta={(\mu_{n}-\mu_{p})}/{\sqrt{\sigma_{n}^{2}+\sigma_{p}^{2}}}$; (ii) Z prime factor $Z^{'}=1-{3(\sigma_{p}+\sigma_{n})}/{|\mu_{p}-\mu_{n}|}$; and (iii) signal-to-background ratio (S/B = ${\mu_{p}}/{\mu_{n}}$), where *µ* and *σ* are mean and standard deviation, and *p* and *n* indicate positive and negative controls (PLA conducted without the BIN1 primary antibody or without the Mouse-minus probe). The optimal parameter set for analysis script was determined to be the one with the highest β factor (β ≥ 2), provided that it produced S/B of at least 10. Additional rounds of parameter optimization were performed when necessary.

#### Full plate analysis and identification of hits

Full plates were analyzed in Columbus with the corresponding optimal analysis script. For each well, Tau area, MAP2 area, and PLA spot area within Tau area were corrected for local bias by normalizing the raw values by the ratio of the local median (median of the surrounding wells in the 5×5 neighborhood) to the plate median (excluding edge wells), *i.e.*, $corrected value={raw value}/\left( \frac{local median}{plate median} \right)$. For each plate, compounds affecting network quality, defined as not having Tau area or Tau:MAP2 area ratio within median ± 3 median absolute deviations (MAD), were excluded (Fig. S6). Edge wells were not taken into account when calculating median and MAD. Corrected PLA:Tau area ratio for each well was then normalized by the mean of non-excluded wells. Finally, normalized, corrected PLA:Tau area ratio obtained from 3 screens were pooled to calculate mean and SEM for each compound that were not excluded from at least 2 screenings (Fig. 5b). Compounds potentially affecting BIN1-Tau interaction were determined as those belonging to the top or bottom 5% tiers (Fig. 5d).

**Validation of selected compounds**

Hit validation was performed by generating dose-response curves for selected compounds to identify specific effects on BIN1-Tau interaction. Since several of the selected compounds had multiple protein targets at 10 µM concentration (as used in the HCS) dose-response experiments were designed to determine if the effects were specific and/or to identify relevant targets. Dose-response experiments were performed for 72 compounds from the top and bottom 5%, which gave similar results in all 3 screens. Compounds were diluted four log scales to obtain a dose-response curve (10 nM, 100 nM, 1 µM and 10 µM) and each compound and concentration was tested in three separate plates under identical conditions as in HCS. Script optimization, plate validation, plate analysis, and well correction and exclusion steps were executed as described above. The DMSO-normalized mean of each compound at 10 µM was compared with the screen result (also at 10 µM), and compounds that had similar effects in both sets of experiments were retained for further analysis. For each compound, dose-response curves were fit with 4-parameter or 3-parameter (where Hill slope is 1) nonlinear regression models, based on the extra sum-of-squares F test using GraphPad Prism 7 (La Jolla, CA), which allowed the calculation of the half maximal effective concentration (EC_50_) value.

**K_d_ determination using NMR data**

The ^1^H,^15^N combined chemical shift changes, ∆δ(^1^H,^15^N), were calculated using the following equation: ∆δ(H,N) = (∆δ^2^_H_ + ∆δ^2^_N_ × 0.159)^1/2^, where Δδ_H_ and Δδ_N_ are the chemical shift (δ) changes for ^1^H and ^15^N, respectively. Dissociation constants were obtained by fitting the chemical shift perturbation data to the following equation: δ_obs_ = Δδ_max_ (a + b + K_d_ – ((a + b + K_d_)^2^ – 4ab)^1/2^) / 2a, where Δδ_obs_ is the weighted average of the chemical shifts in the free and bound states and Δδ_max_ is the maximal signal change upon saturation (bound state). K_d_ is the dissociation constant, *a* and *b* are the total peptide and BIN1 SH3 concentrations, respectively. K_d_ was calculated based on chemical shift perturbations of each resonance with ∆δ(H,N) > 0.01 ppm when comparing the bound and free states, and averaged.

**G-ratio calculation**

Internal and external diameters of myelin sheats in electron microscopy images were measured using Image J. External myelin diameter was divided by the internal diameter (n = 2 mice per genotype; ~50 axons per mouse). To avoid bias due to axons projecting at different angles through the tissue section, minimal Feret diameter was consistently used.

**Supplementary Results**

**Myelin alterations in the fornix**

The degree of fornix alteration is correlated with memory impairments. Coronal visualization of the fornix at 18 months in hTau and hTau;Tg*BIN1* mice was performed using electron microscopy. *MAPT* overexpression is associated with myelin abnormalities. Indeed, most nerve fibers in the hTau mice presented multiple myelin rings (Fig. S27, arrowheads); however, a significant rescue of this phenotype was observed in hTau;Tg*BIN1* mice as evidenced by the recovery of the g-ratio of myelinated fibers (Fig. S27g). The overexpression of BIN1 alone did not induce any myelin abnormalities (Fig. S28). Thus, the memory impairments observed in the behavioral analyses of the hTau mice may be also associated with myelin disorganization in the fornix, and were rescued upon BIN1 overexpression. Finally, as spatial memory was perturbed in hTau mice, ultrastructural analysis of proximal axons projecting from the CA3 was also conducted *via* electron microscopy. No obvious ultrastructural defects were observed and, notably, the microtubule network appeared well aligned and distributed inside the nerve fibers in hTau*,* Tg*BIN1*, and hTau;Tg*BIN1* mice (Fig. S29).

**Supplementary Tables**

**Table S1.** Primer sequences

| **Primer** | **5’🡪 3’** |
| --- | --- |
| Mapt KI WT UP | CTCAGCATCCCACCTGTAAC |
| Mapt KI WT DW | CCAGTTGTGTATGTCCACCC |
| Mapt KI Tg UP | AAGTTCATCTGCACCACCG |
| Mapt KI Tg DW | TGCTCAGGTAGTGGTTGTCG |
| TgMAPT (mut) UP | ACTTTGAACAGGATGGCTGAGCCC |
| TgMAPT (mut) DW | CTGTGCATGGCTGTCCACTAACCTT |
| TgMAPT (WT) UP | CTAGGCCACAGAATTGAAAGATCT |
| TgMAPT (WT) DW | GTAGGTGGAAATTCTAGCATCATCC |
| TgBIN1 UP | CGAGGCCTGCGCCGCGATGGC |
| TgBIN1 DW | CGCAGCCTGGGGACCTCGAAG |

**Table S2.** Antibodies used in brain slices

| **Antibody** | **Reference** |
| --- | --- |
| Tau [E178] | Abcam, ab32057 |
| BIN1 [C99D] | Sigma, B9428 |
| Phospho Tau Thr231 [AT180] | Thermo Scientific, MN1040 |
| Phospho Tau Ser202 [AT8] | Thermo Scientific, MN1020 |

**Table S3.** Demographic details of the neuropathological cohort.

| **Individual** | **Braak stage** | **Gender** | **Age at death** | **Post-mortem delay (h)** | **Brain weight (g)** | **Neuropathological diagnosis** |
| --- | --- | --- | --- | --- | --- | --- |
| 1 | 0 | M | 61 | 7 | 900 | Non-AD |
| 2 | 0 | F | 52 | 8 | 1190 | Non-AD |
| 3 | 1 | F | 82 | 7 | 950 | AD |
| 4 | 1 | M | 84 | 7 | 1130 | Non-AD |
| 5 | 2 | F | 84 | 5 | 1100 | AD |
| 6 | 2 | F | 82 | 4 | 1110 | AD |
| 7 | 3 | F | 76 | 4 | 990 | AD |
| 8 | 3 | F | 92 | 18 | 1060 | Non-AD |
| 9 | 4 | F | 76 | 24 | 1165 | AD |
| 10 | 4 | F | 85 | 4 | 1070 | AD |
| 11 | 5 | F | 85 | 4 | 800 | AD |
| 12 | 5 | M | 80 | 5 | 1000 | AD |
| 13 | 6 | F | 78 | 5 | 995 | AD |
| 14 | 6 | F | 85 | 4 | 1035 | AD |
| 15 | 0 | F | 86 | 3 | 1050 | Non-AD |
| 16 | 0 | M | 60 | 48 | 1515 | Non-AD |
| 17 | 1 | M | 83 | 13 | N/A | Non-AD |
| 18 | 1 | F | 87 | 5 | 1065 | Non-AD |
| 19 | 2 | F | 80 | 96 | 1050 | Non-AD |
| 20 | 2 | M | 79 | 34 | 1370 | Non-AD |
| 21 | 3 | M | 81 | 4 | 1360 | AD |
| 22 | 3 | F | 100 | 12 | 940 | AD |
| 23 | 4 | F | 79 | 4 | 1105 | AD |
| 24 | 4 | F | 85 | 24 | 1020 | AD |
| 25 | 5 | F | 97 | 4 | 965 | AD |
| 26 | 5 | F | 76 | 3 | 900 | AD |
| 27 | 6 | F | 82 | 7 | 740 | AD |
| 28 | 6 | F | 74 | 6 | 1000 | AD |

**Supplementary Figures:**

**
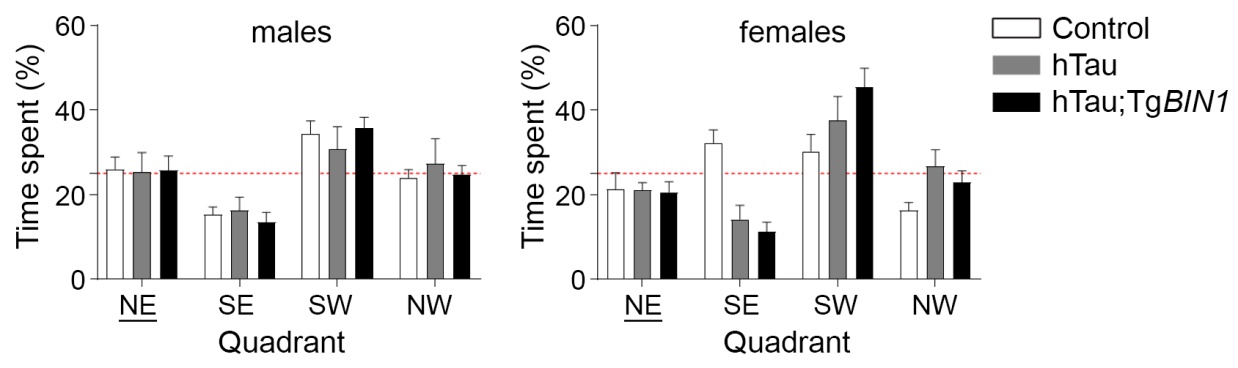
**

**Fig. S1** Probe test without platform with 15-month-old hTau and hTau;Tg*BIN1* males, performed 24 h before the training session. Dashed line represents chance. Data represent mean ± SEM for each quadrant (control, n=11; hTau, n=10; hTau;Tg*BIN1*, n=13). Underlined quadrant marks original platform location. One-sample t-test compared to chance at 25%.

**
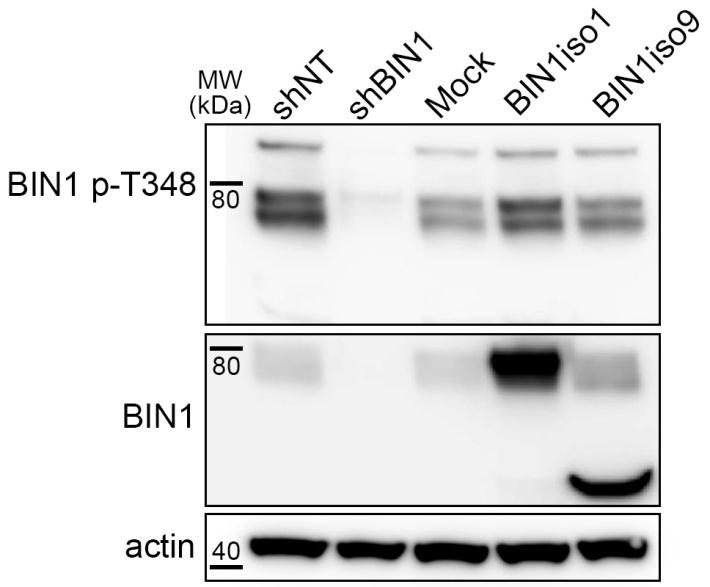
**

**Fig. S2** Specificity of BIN1 p-T348 antibody to the neuronal isoforms of BIN1 in PNC. Western blot of neurons transduced with shNT, shBIN1, Mock, BIN1iso1 (containing T348), and BIN1iso9 (without the CLAP domain or T348) constructs. BIN1 p-T348 signal is modulated as a function of BIN1iso1 under- and overexpression. No BIN1 p-T348 signal was detected at the molecular weight of BIN1iso9, while endogenous BIN1iso1 was seen in all but the shBIN1-expressing neurons.


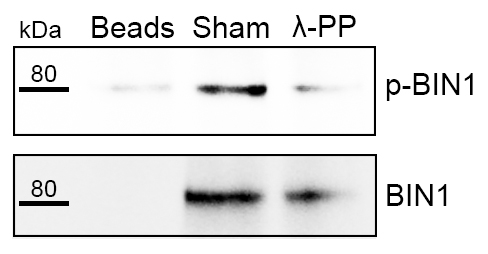


**Fig. S3** Immunoprecipitation using primary neurons. IP performed with total BIN1 antibody (99D) in PNC shows the specificity of p-T348 antibody towards phosphorylated BIN1. λ-PP treatment caused 19.4-fold decrease in p-BIN1:BIN1 ratio after background (beads signal) subtraction. p-T348 signal detected for the λ-PP treatment condition is similar to the beads-only condition, which constitutes the background noise inherent to this technique.

**
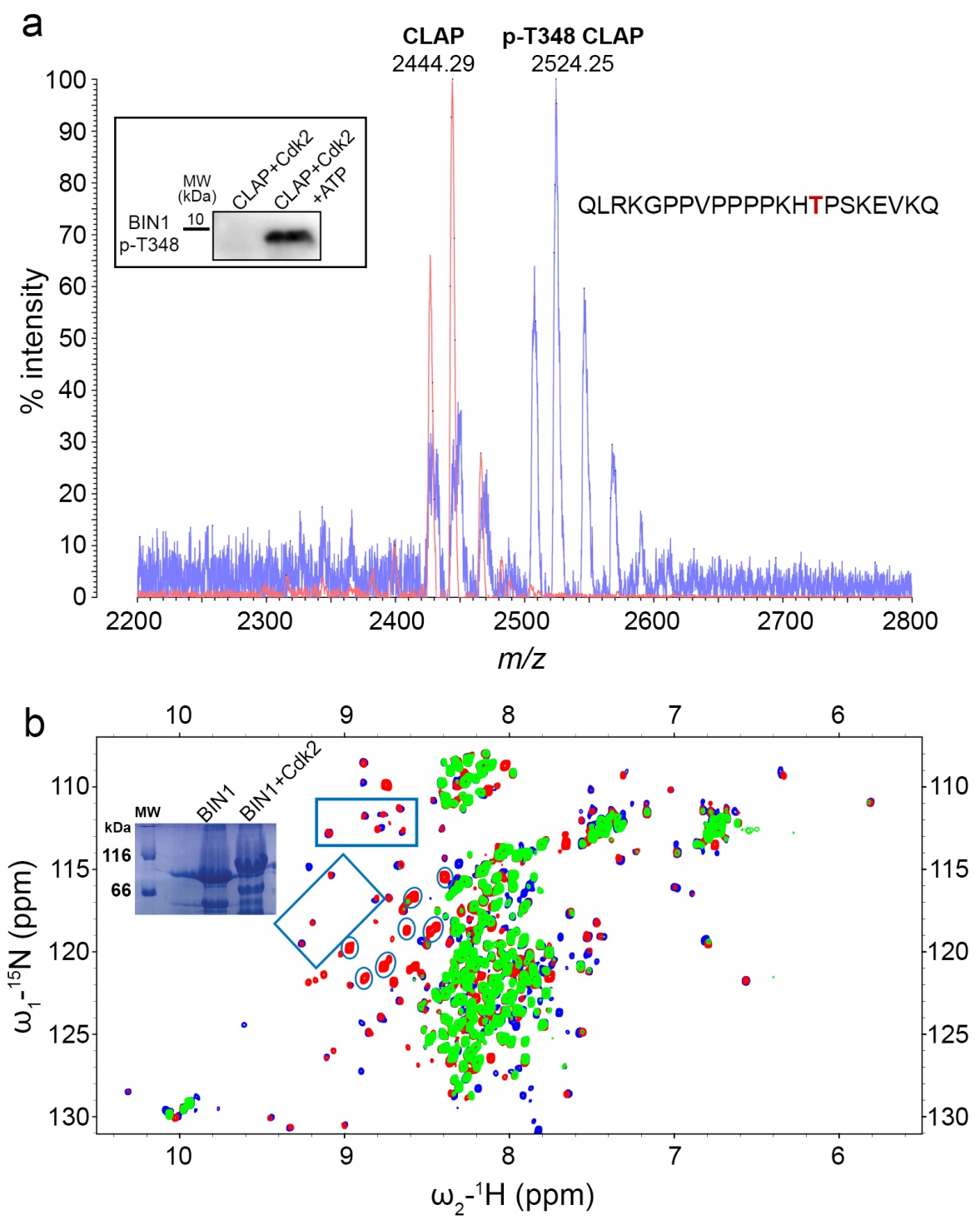
**

**Fig. S4** NMR measurements confirm that Cdk2 phosphorylates BIN1 CLAP at T348. **a.** Maldi-TOF analysis of CLAP (334-355) peptide before (red) and after (blue) incubation with Cdk2/CycA3 kinase. Incubation of the peptide with the kinase (molar ratio 1/100) at 37°C for 3 h, in the presence of 2 mM ATP, 2.5 mM MgCl_2_, 2 mM EGTA, 2 mM DTT, 30 mM NaCl and protease inhibitors in 50 mM HEPES, pH 8.0, resulted in a mass increase compatible with the incorporation of one phosphate group. T238 is the only Pro-directed site compatible with the kinase specificity in the peptide. Inset: BIN1 p-T348 antibody recognizes the BIN1 CLAP (334-355) peptide upon Cdk2 phosphorylation. **b.** ^1^H-^15^N HSQC spectra of BIN1Iso1-CLAP-T348E protein (blue), Cdk2-phospho-BIN1Iso1 (superimposed in red) and BIN1Iso1 protein (superimposed in green). Boxed resonances, superimposed in the blue and red spectra, correspond to some of the resonances of the BIN1-SH3 domain, and are only detected in the presence of T348 phosphorylation or the T348E mutation of the CLAP domain (see also Fig. S22). Circled resonances, only detected in the red spectrum (or Cdk2-phospho-BIN1) correspond to resonances with typical H-N chemical shift values for pS/pT residues. Inset: Multiple sites of Cdk2-BIN1 were thus modified in the conditions of this assay, as also shown by the characteristic gel-shift observed by SDS-PAGE.

**
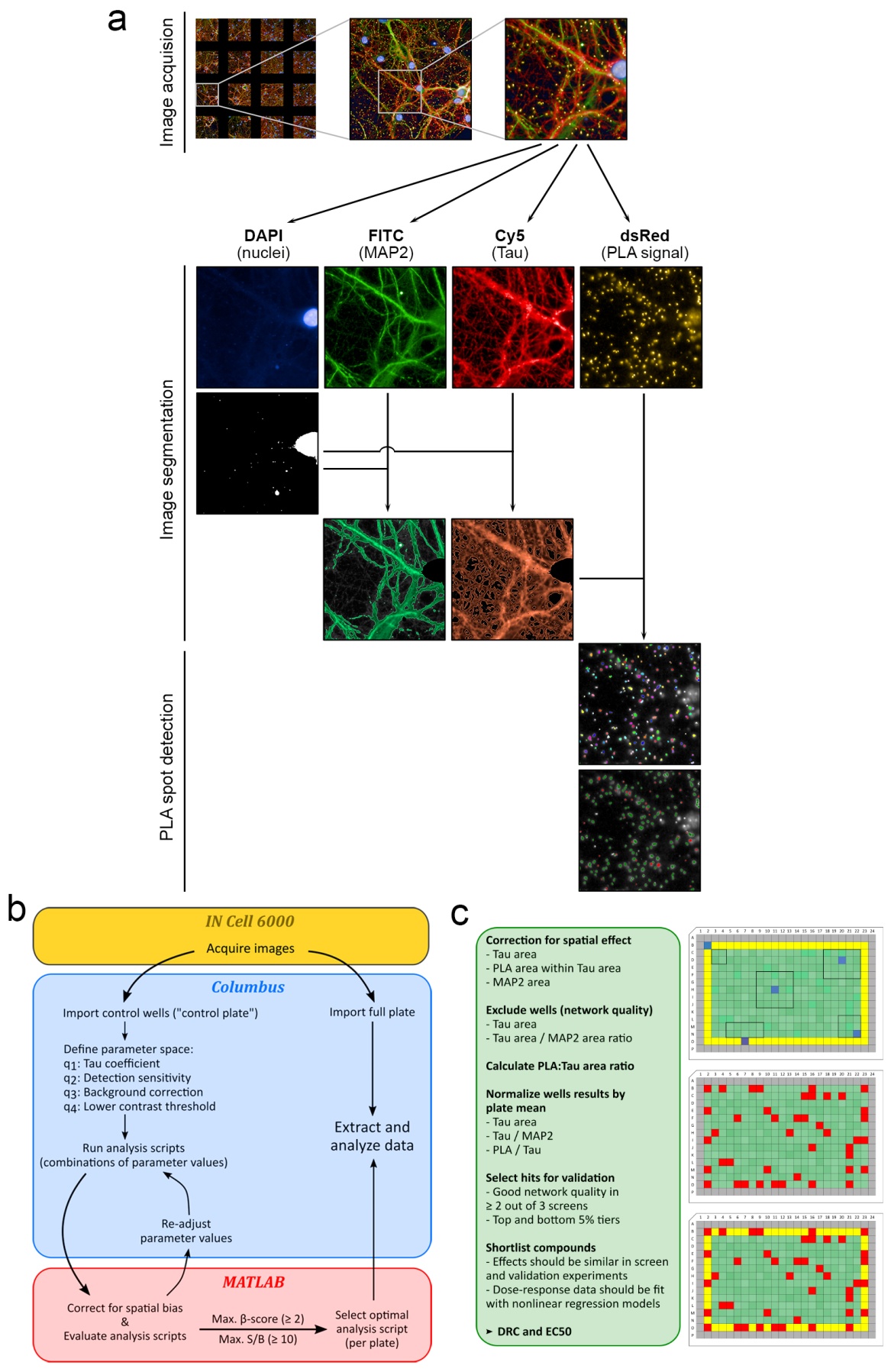
**

**Fig. S5** HCS image analysis and quantification procedures. **a.** Details of image acquisition, segmentation and PLA detection processes: Acquisition of 16 fields per well in four wavelengths using IN Cell Analyzer 6000; exclusion of somatic regions from FITC and Cy5 channels and delimitation of MAP2 and Tau areas in the Columbus software; detection of PLA spots in non-somatic Tau areas and their filtering based on area and contrast (green spots in the last image). **b.** Schematic showing the iterative process for selecting the optimum analysis script separately for each plate. **c.** Demonstration of data analysis in a representative plate layout. Yellow wells are used to correct for spatial bias. Blue wells and the corresponding 5×5 neighborhood (black outlines) illustrate the correction for local bias. Red wells were excluded based on network quality and the remaining (green) wells were used to calculate plate means for normalization.

**
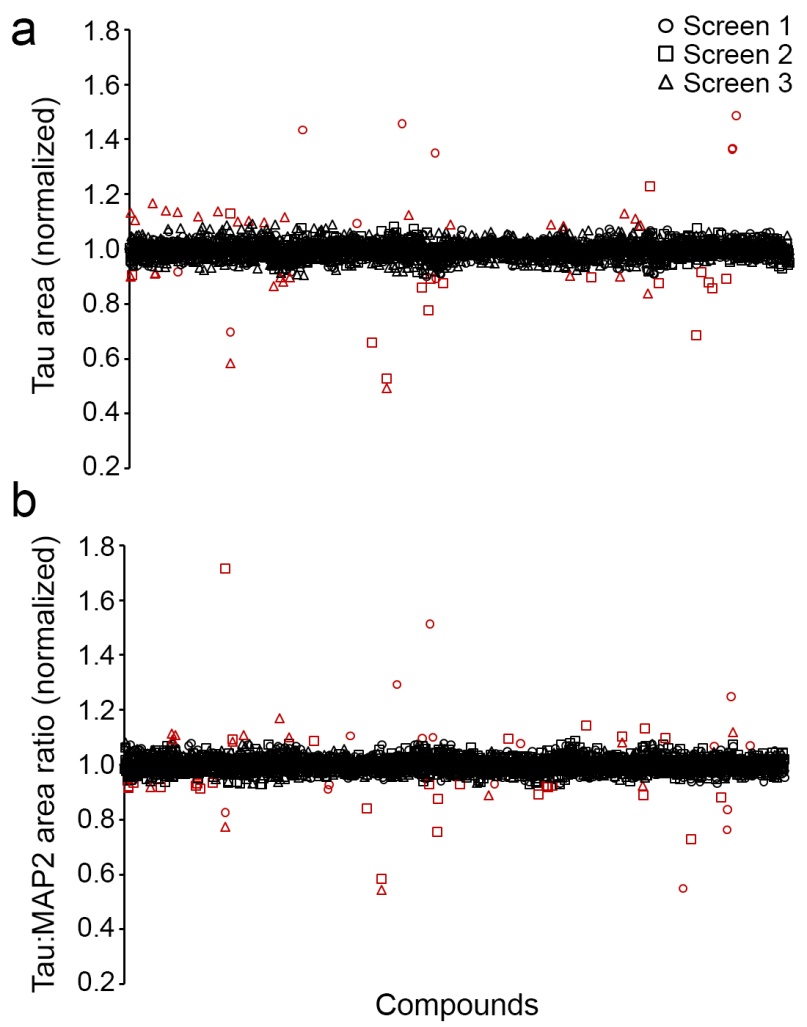
**

**Fig. S6** Distribution of Tau area (**a**) and Tau:MAP2 area ratio (**b**) of all 1,126 compounds in the three screenings, after plate-by-plate normalization (excluded data points shown in red).

**
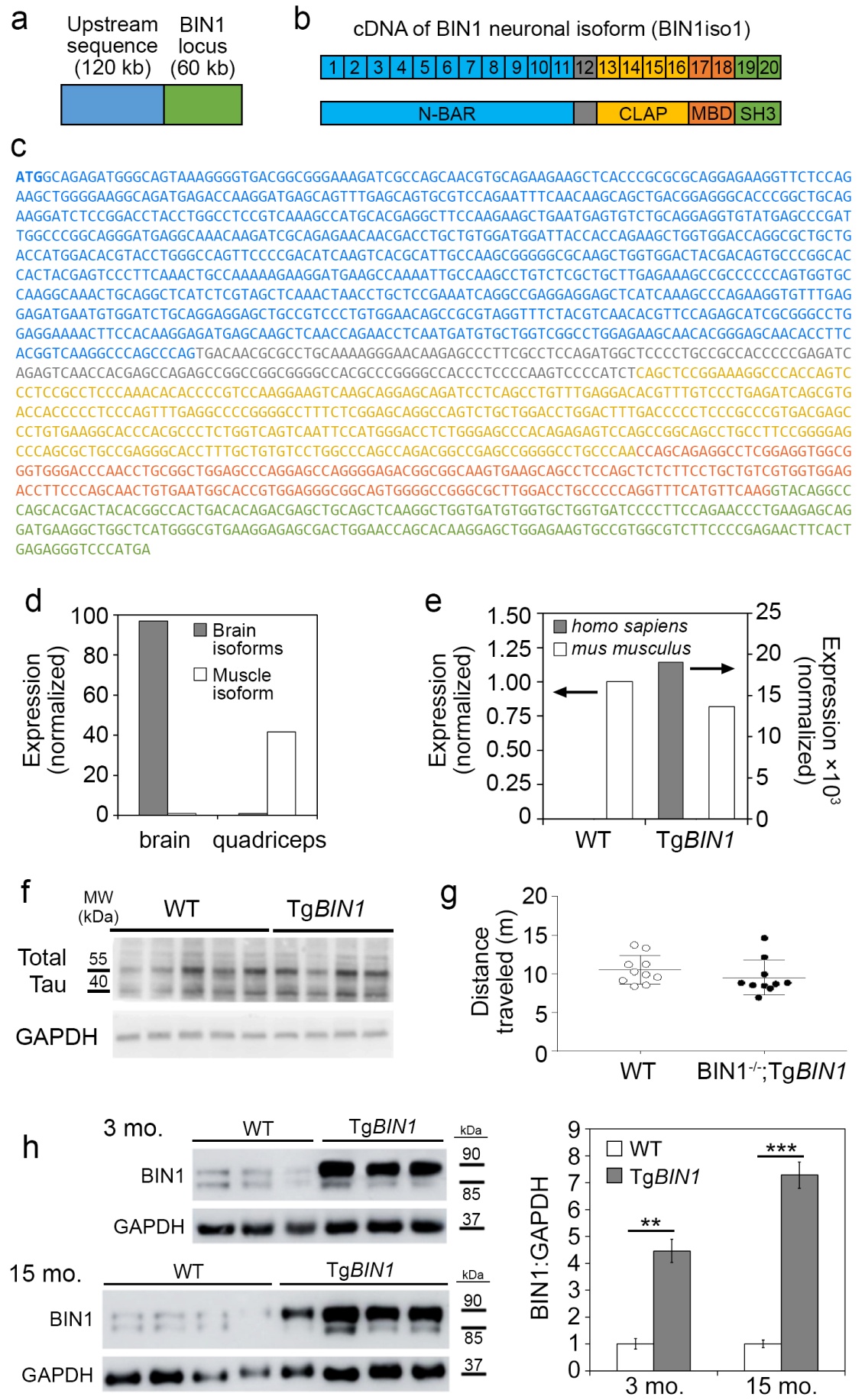
**

**Fig. S7** MAPT and BIN1 expression in the Tg*BIN1* mouse. **a.** Drawing of the BAC RP11-437K23 encompassing the *BIN1* locus and upstream sequences. **b.** RNA was extracted from hippocampus of Tg*BIN1* mice, reverse-transcribed, cloned and sequenced. Human exons present in hippocampus and corresponding protein domains are displayed with corresponding colors. BAR, BIN-Amphiphysin-Rvs; CLAP, Clathrin and AP2 binding domain; MBD, Myc binding domain; SH3, Src homology domain. **c.** Isoform 1 was the main human BIN1 isoform detected, whose sequence is shown. **d.** Quantification of neuronal and muscular BIN1 isoforms by RT-qPCR from brain and quadriceps of Tg*BIN1* mouse. **e.** Quantification of total murine Bin1 and human BIN1 RNA by RT-qPCR from brain of WT and Tg*BIN1* mice. **f.** Western blots of total Tau in the brains of WT and Tg*BIN1* mice. **g.** Distance traveled by WT and BIN1^-/-^;Tg*BIN1* mice in the open field test. **h.** BIN1 expression in WT and TgBIN1 mice at 3 and 15 months. Student’s *t*-test; ** p < 0.01, *** p < 0.001.

**
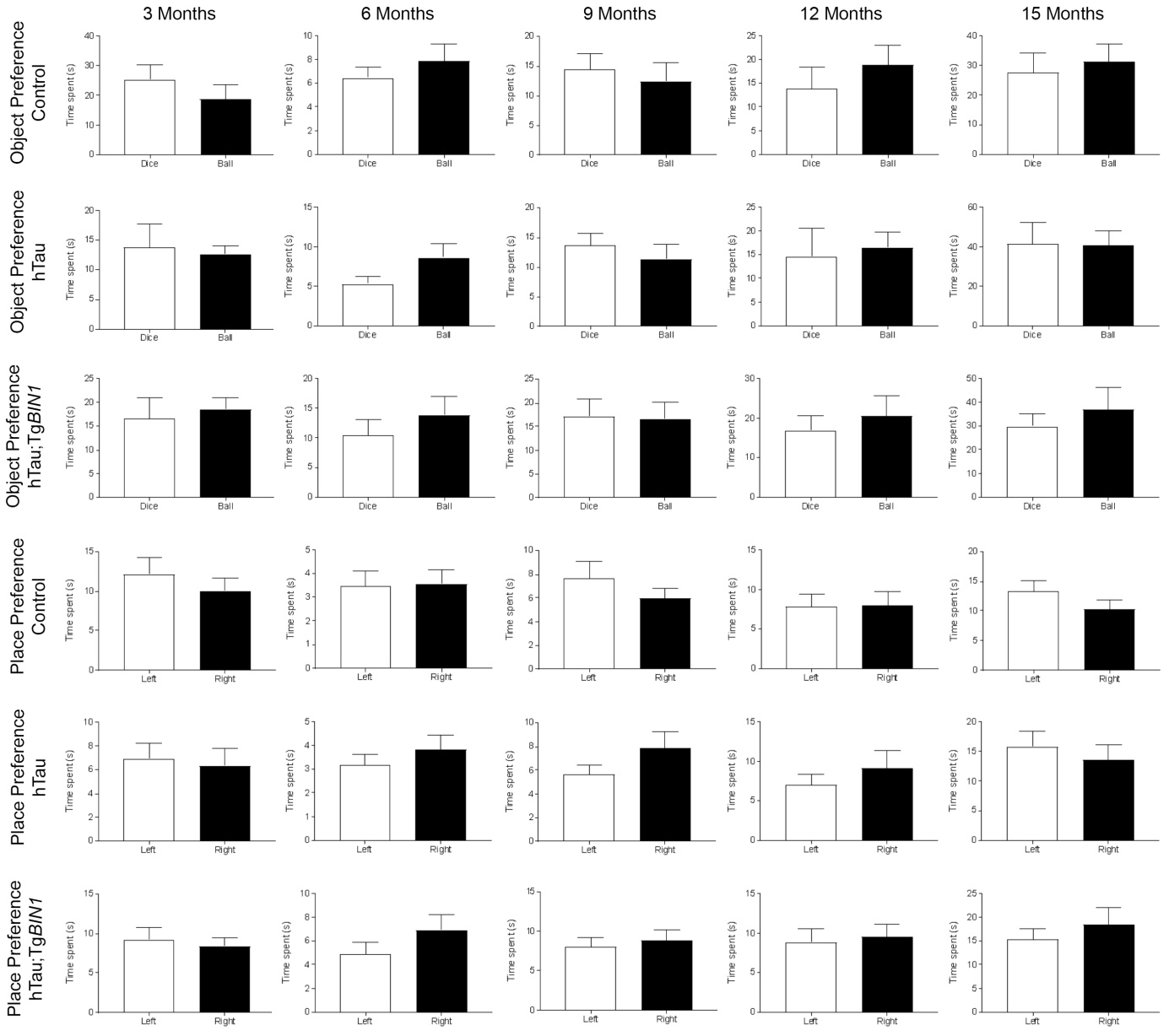
**

**Fig. S8** Validation of lack of object preference. Percentage of time spent by the 3-, 6-, 9-, 12-, or 15-month-old control, hTau and hTau;Tg*BIN1* mice with objects located in the right or left positions, during the acquisition phase.

**
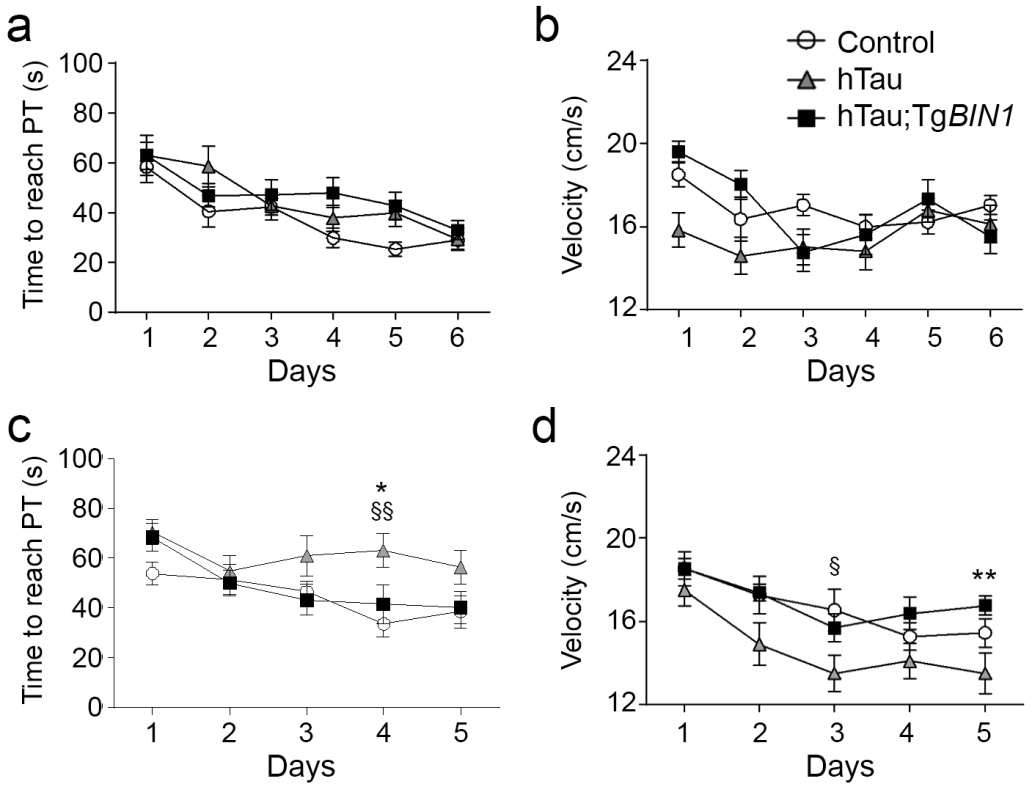
**

**Fig. S9** Spatial and long-term memory in 12- and 15-month-old Tg*BIN1* males assessed with Morris water maze. **a.** Time to reach the platform (PT) at 12 months. **b.** Velocity during task acquisition. **c.** Time to reach the platform (PT) at 15 months. **d.** Velocity during task acquisition. Data represent mean ± SEM for consecutive days of acquisition (control n=11; hTau n=11; hTau;Tg*BIN1* n=13). Two-way ANOVA followed by Bonferroni *post hoc* test at each day of acquisition. § p < 0.05, §§ p < 0.01 for control vs hTau. * p < 0.05, ** p < 0.01 for hTau vs hTau;Tg*BIN1*.

**
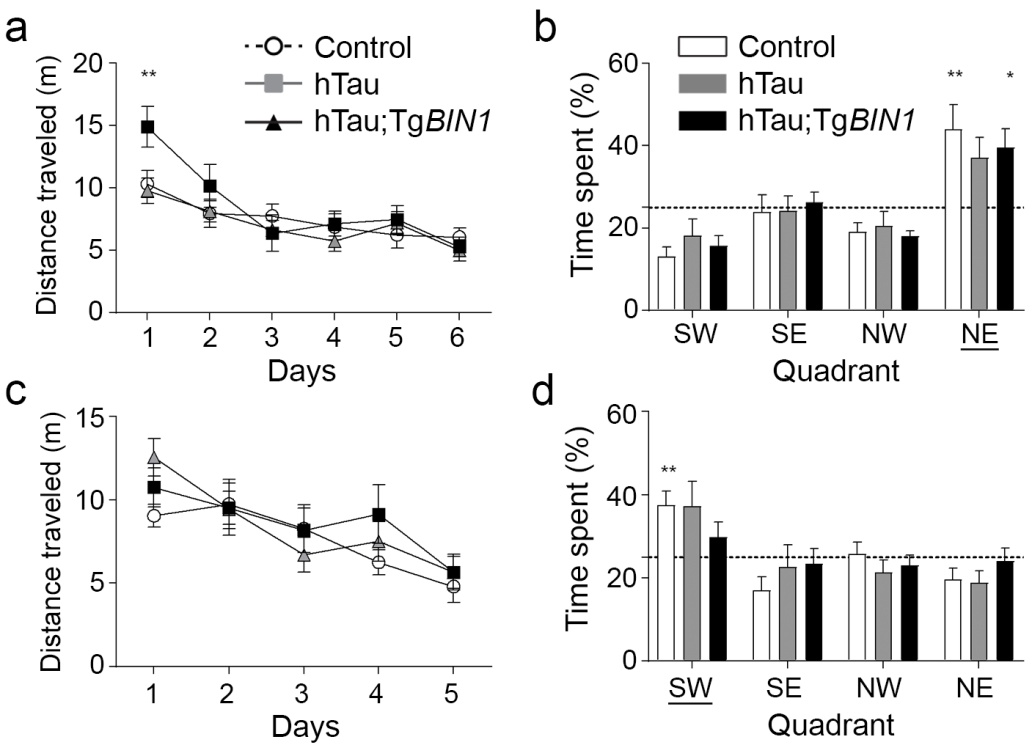
**

**Fig. S10** Long-term memory deficits due to *MAPT* overexpression in hTau females is rescued by BIN1 overexpression at 12 but not 15 months. Spatial and long-term memory in 12- and 15-month-old control, hTau and hTau;Tg*BIN1* mice assessed with Morris water maze. **a.** Distance traveled to reach the platform at 12 months. Data represent mean ± SEM for consecutive days of acquisition (control, n=11; hTau, n=9; hTau;Tg*BIN1*, n=11). **b.** Probe test without platform at 12 months, performed 24 h after the last training session. Dashed line represents chance. Data represent mean ± SEM for each quadrant (control, n=11; hTau, n=9; hTau;Tg*BIN1*, n=11). Underlined quadrant marks original platform location. **c.** Distance traveled to reach the platform at 15 months. Data represent mean ± SEM for consecutive days of acquisition (control, n=11; hTau, n=9; hTau;Tg*BIN1*, n=11). **d.** Probe test without platform at 15 months, performed 24 h after the last training session. Dashed line represents chance. Data represent mean ± SEM for each quadrant (control, n=11; hTau, n=9; hTau;Tg*BIN1*, n=11). Underlined quadrant marks original platform location. One sample t-test compared to chance at 25% for the time spent in quadrants, *p<0.05, **p<0.01. Two-way ANOVA for the distance travelled, **p<0.01.


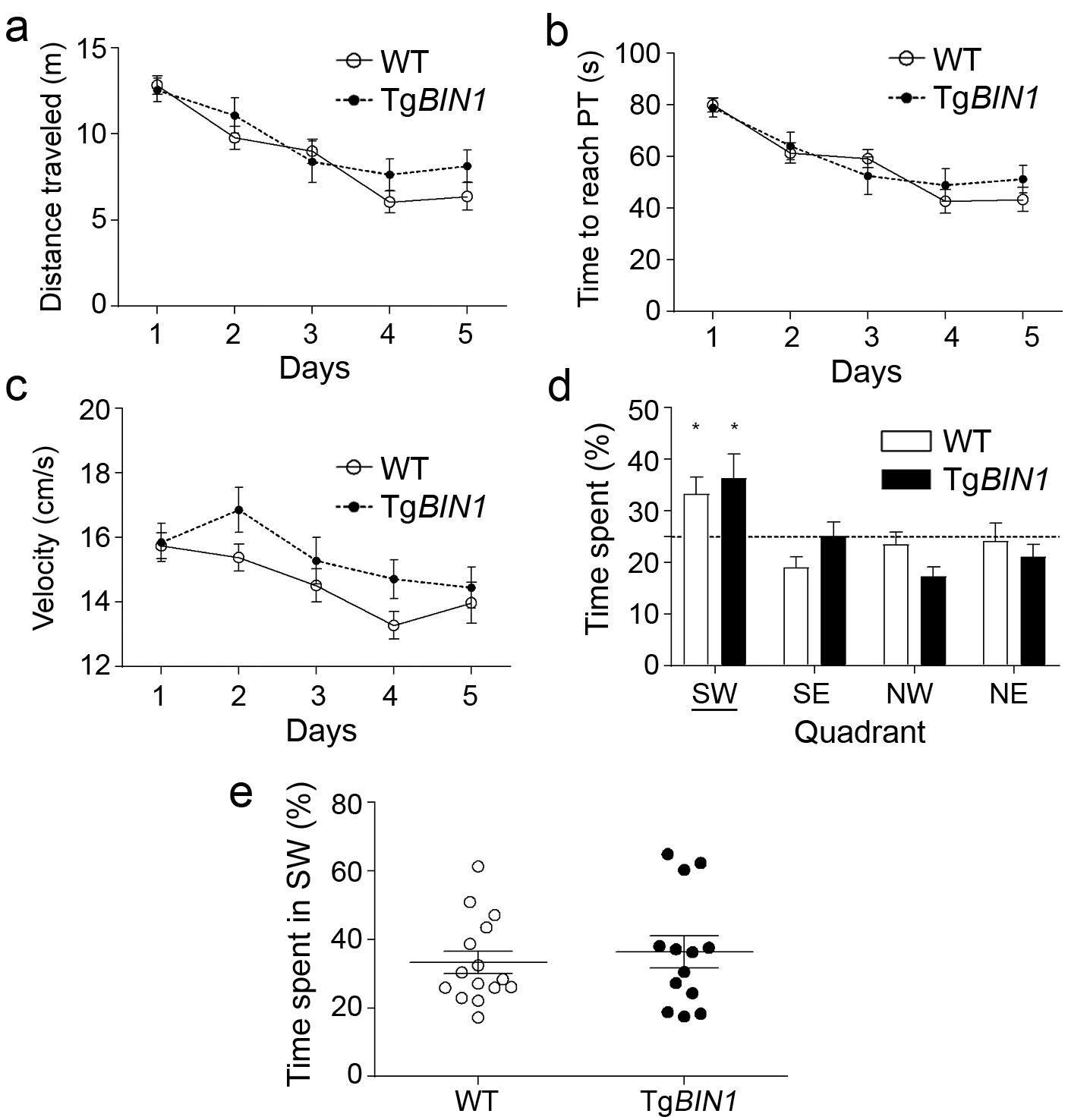


**Fig. S11** *BIN1* overexpression does not affect long-term memory. Spatial and long-term memory in 15-month-old Tg*BIN1* males assessed with Morris water maze. **a.** Distance traveled to reach the platform. Data represent mean ± SEM for consecutive days of acquisition (WT, n=15; Tg*BIN1*, n=13). **b.** Time to reach the platform (PT). Data represent mean ± SEM for consecutive days of acquisition (WT, n=15; Tg*BIN1*, n=13). **c.** Velocity during task acquisition. Data represent mean ± SEM for consecutive days of acquisition (WT, n=15; Tg*BIN1*, n=13). **d.** Probe test without the platform, performed 24 h after the last training session. Dashed line represents chance. Data represent mean ± SEM for each quadrant (WT, n=15; Tg*BIN1*, n=13). Underlined quadrant marks original platform location. One sample t-test compared to chance at 25%; * p < 0.05. **e.** Dot plot ± SEM from the showing the baseline performances of 15-month-old WT and Tg*BIN1* males. Probe test without the platform, performed 24 h after the last training session. Dashed line represents chance. No statistical significance according to Student’s *t*-test (WT, n=15; Tg*BIN1*, n=13).

**
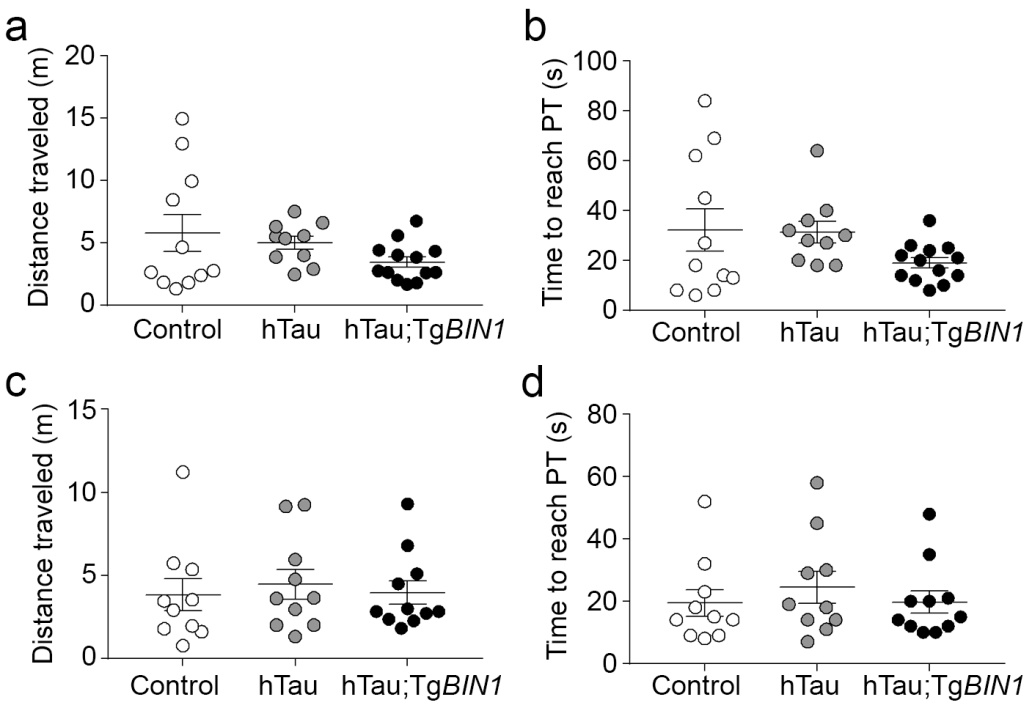
**

**Fig. S12** Absence of visual or motor deficits in 15-month-old males and females for the Morris water maze. **a.** Distance traveled to reach the visible platform (PT) for males (control, n=11; hTau, n=11; hTau;Tg*BIN1*, n=13). **b.** Time to reach the visible platform for males (control, n=11; hTau, n=11; hTau;Tg*BIN1*, n=13). **c.** Distance traveled for females to reach the visible platform (PT; control, n=10; hTau, n=10; hTau;Tg*BIN1*, n=11). **d.** Time to reach the visible platform for females (control, n=10; hTau, n=10; hTau;Tg*BIN1*, n=11). Data represent mean ± SEM. One-way ANOVA followed by Bonferroni *post hoc* test.

**
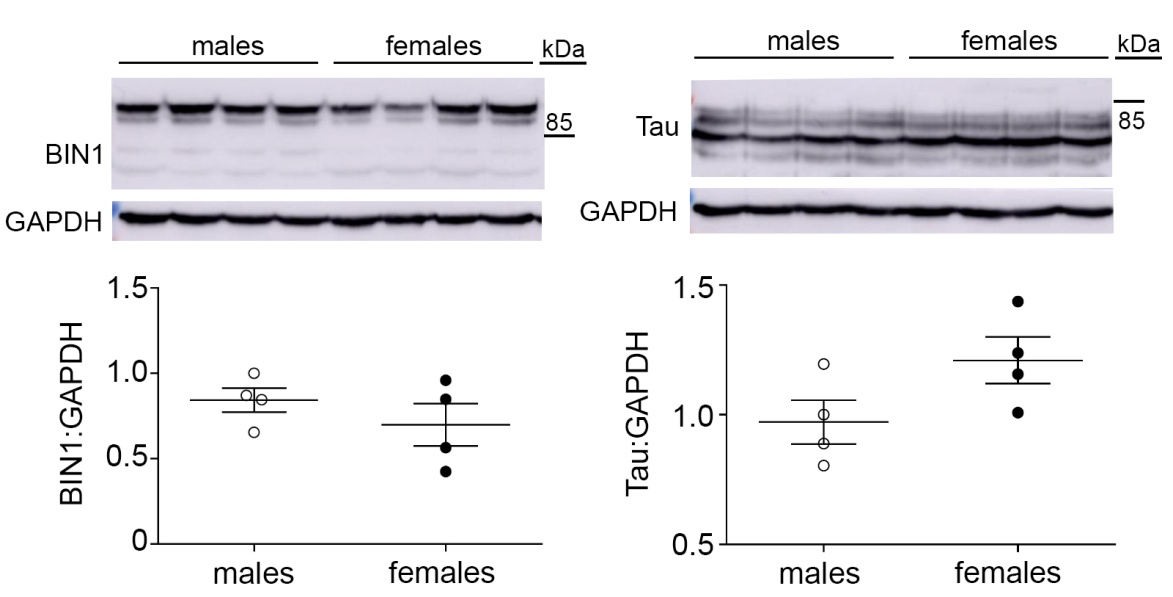
**

**Fig. S13** BIN1 and Tau protein levels in the hippocampi of male and female htau;Tg*BIN1* mice quantified by Western blotting.


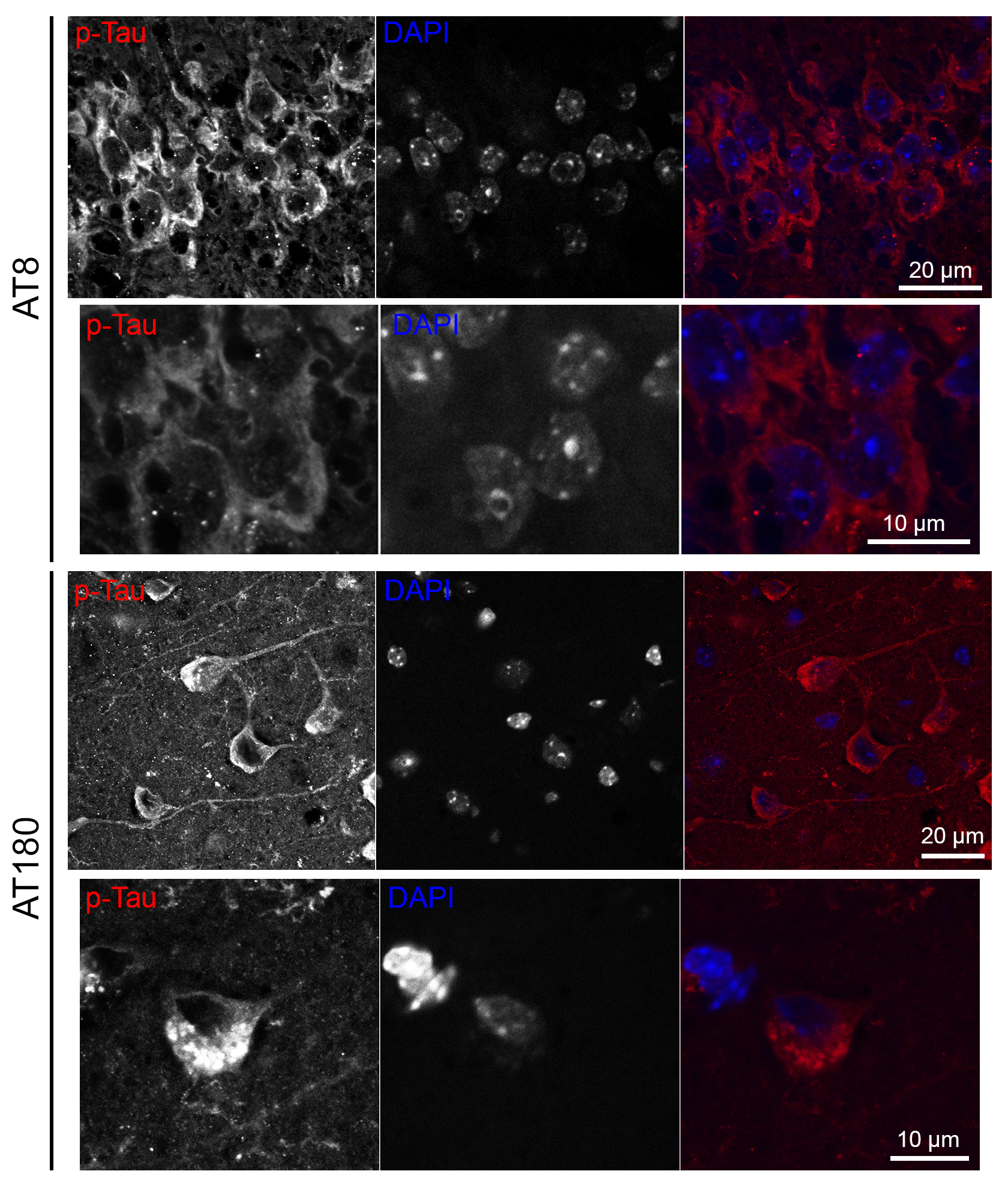


**Fig. S14** Confocal images of hippocampal slices of 18-month-old hTau mice. Tau inclusions exhibit perinuclear and axonal localization when immunostained by AT8 and AT180 antibodies.

**
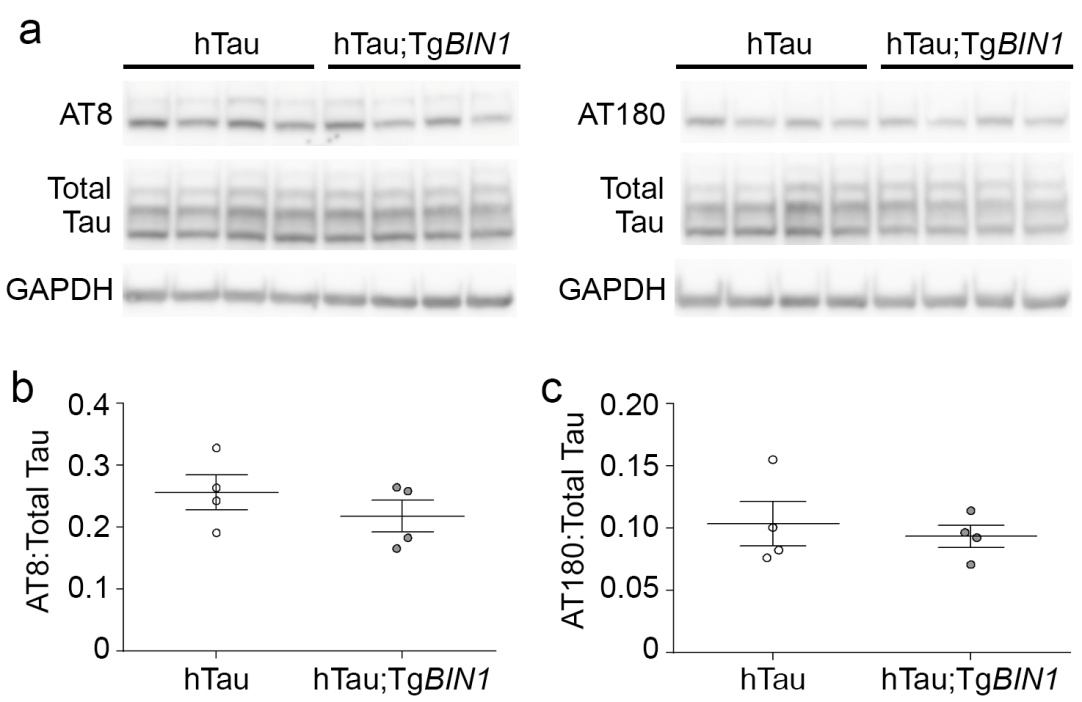
**

**Fig. S15** BIN1 does not impact the levels of soluble phospho-Tau proteins. **a**. Western blots of hippocampal lysates from 18-month-old hTau and hTau;Tg*BIN1* male mice, labeled with antibodies detecting total Tau protein (total Tau), p-Ser202/p-Thr205 Tau (AT8) or p-Thr231 Tau (AT180), and GAPDH. Quantification of phospho-Tau over total Tau signal intensities for AT8 (**b**) and AT180 (**c**) antibodies.

**
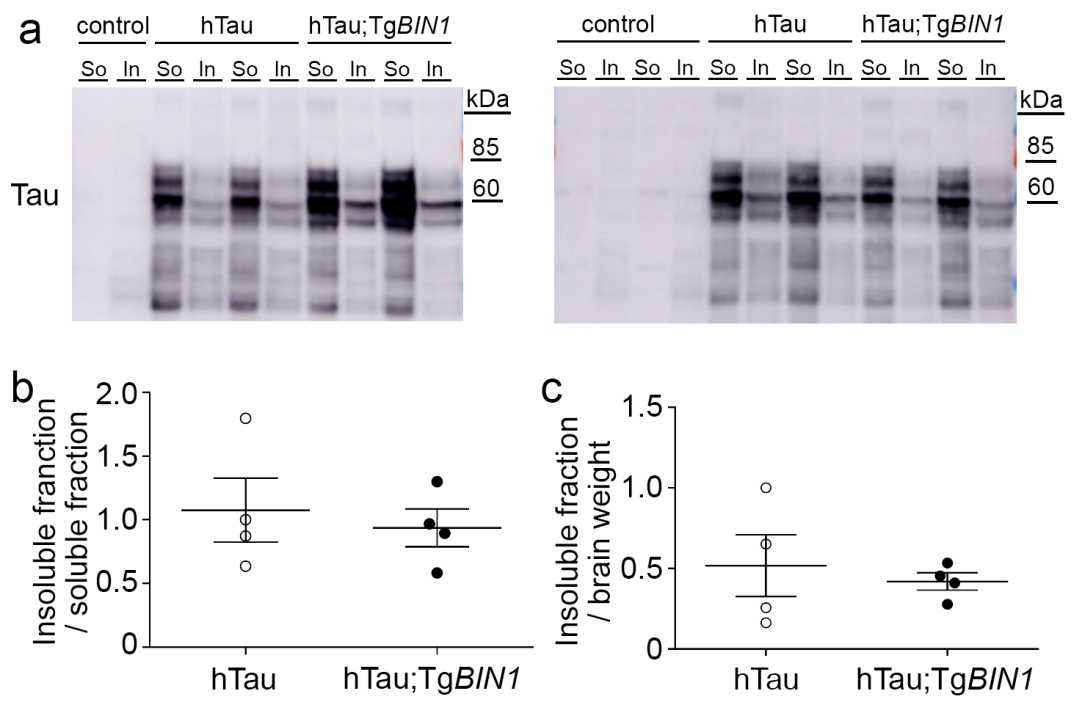
**

**Fig, S16** Western blotting following brain sarkosyl extraction. **a.** Tau immunoblots of control, hTau, and hTAu;Tg*BIN1* mice using anti-tau RD3 (8E6/C11) antibody on the sarcosyl-soluble (So) and sarcosyl-insoluble (In) fractions. **b.** Optical density of the insoluble fraction was normalized either by that of the soluble fraction or by the brain weight.

**
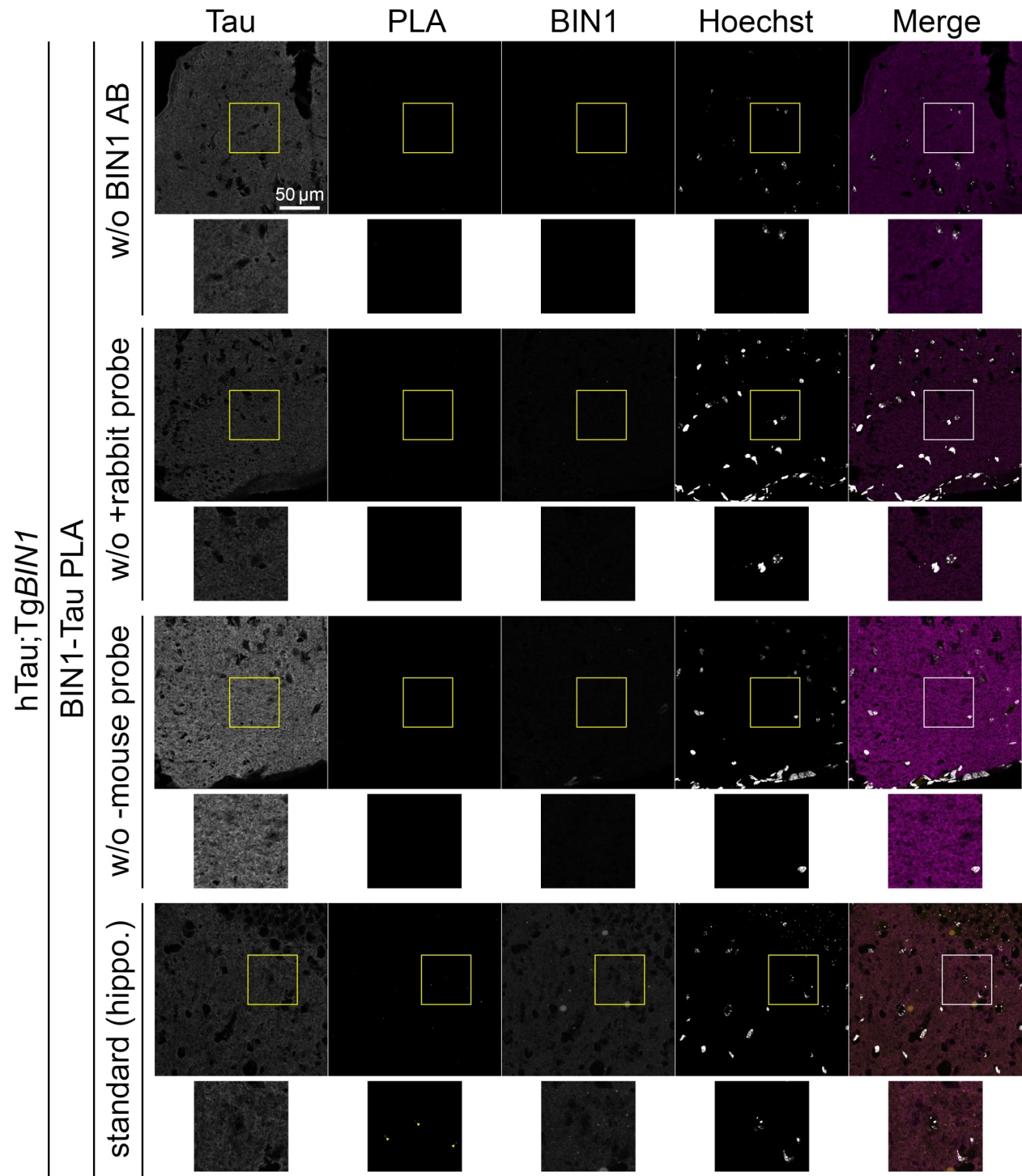
**


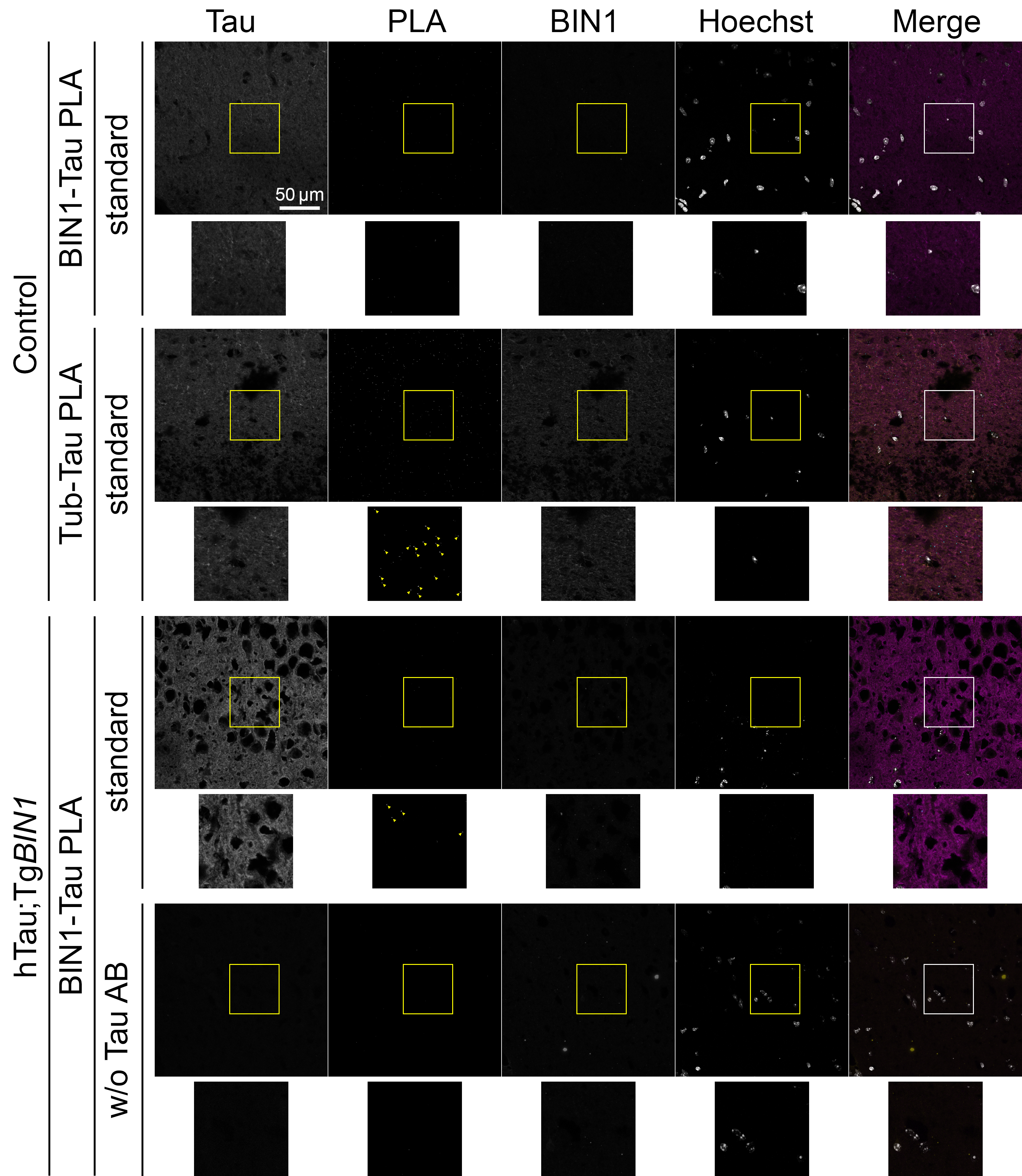


**Fig. S17** Specificity of the BIN1-Tau PLA in mouse brain slices. Tubulin-Tau PLA in control mice was used as a positive control. BIN1-Tau PLA in hTau;TgBIN1 mice was considered as the standard method. Negative controls consisted of the standard method lacking either the Tau or BIN1 antibodies or +rabbit or –mouse PLA probes. The standard method was further optimized for hTau;TgBIN1 hippocampal slices. Boxed regions were 1.95× magnified. Arrowheads indicate true PLA signals. AB: antibody; w/o: without.

**
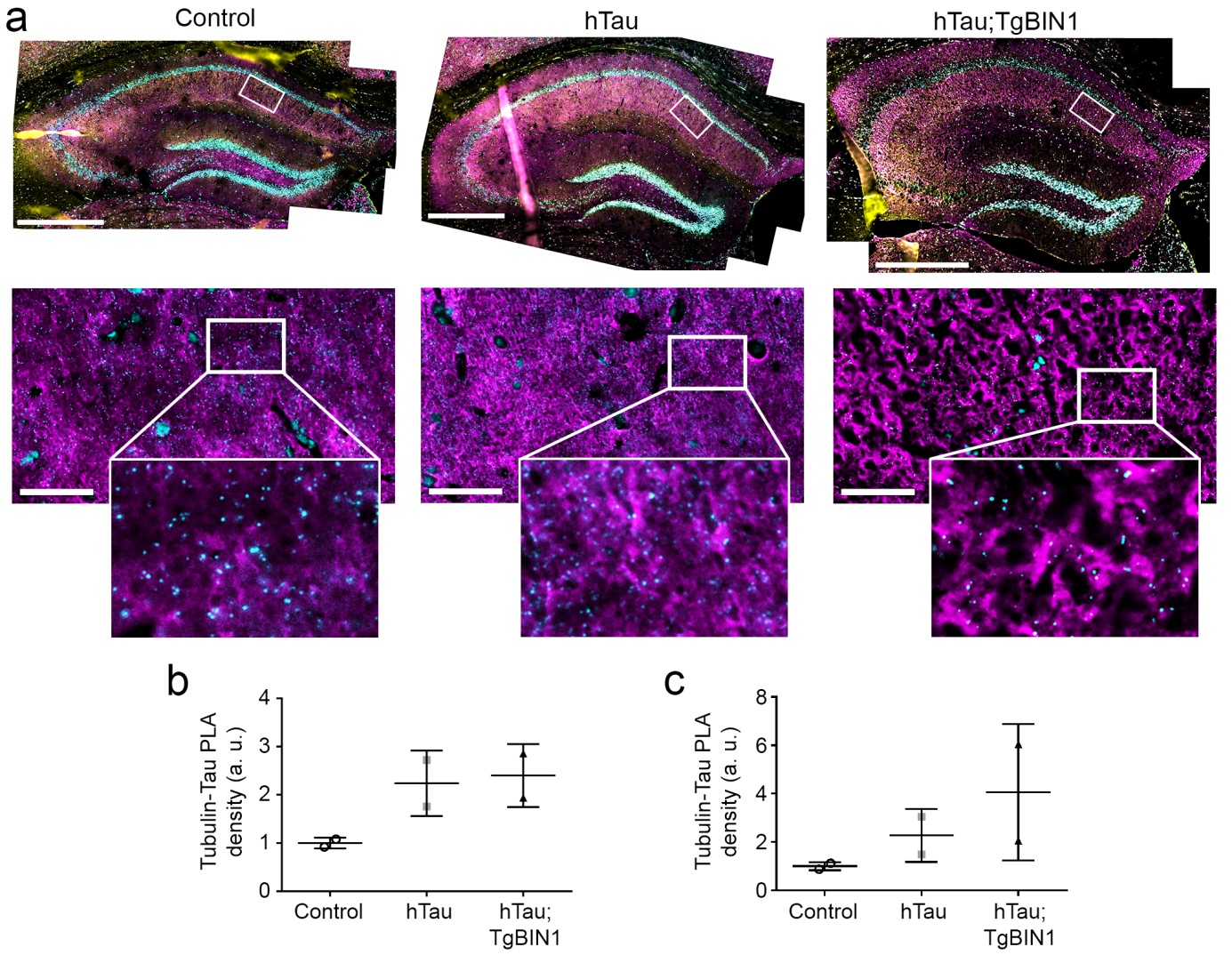
**

**Fig. S18** Tubulin-Tau PLA in brain slices. **a.** Tubulin-Tau PLA (cyan), and Tubulin (yellow), Tau (magenta), and Hoechst (white) stainings in the hippocampi of control, hTau and hTau;Tg*BIN1* males at 18 months. Zoomed areas show PLA and Tau channels only. Scale bars = 500 µm; zooms, 50 µm. **b-c.** Quantification of the Tubulin-Tau PLA density. Data expressed as PLA spot number per tissue area (B) or total PLA spot volume per tissue area (C), normalized with control mean (control, n=2; hTau, n=2; hTau;Tg*BIN1*, n=2).


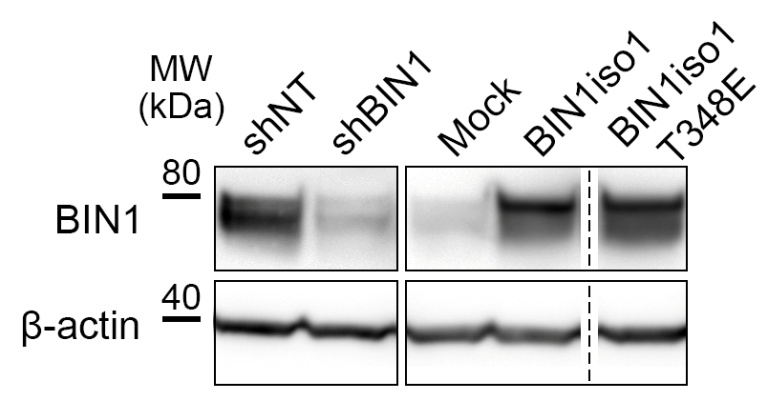


**Fig. S19** Expression of BIN1 after transduction with BIN1 constructs. Representative Western blots showing BIN and β-actin protein levels in PNC at DIV14 after transduction at DIV8. Bands showing shNT and shBIN1 are from the same membrane as the other constructs, but with longer exposure to reveal the shBIN1 band.

**
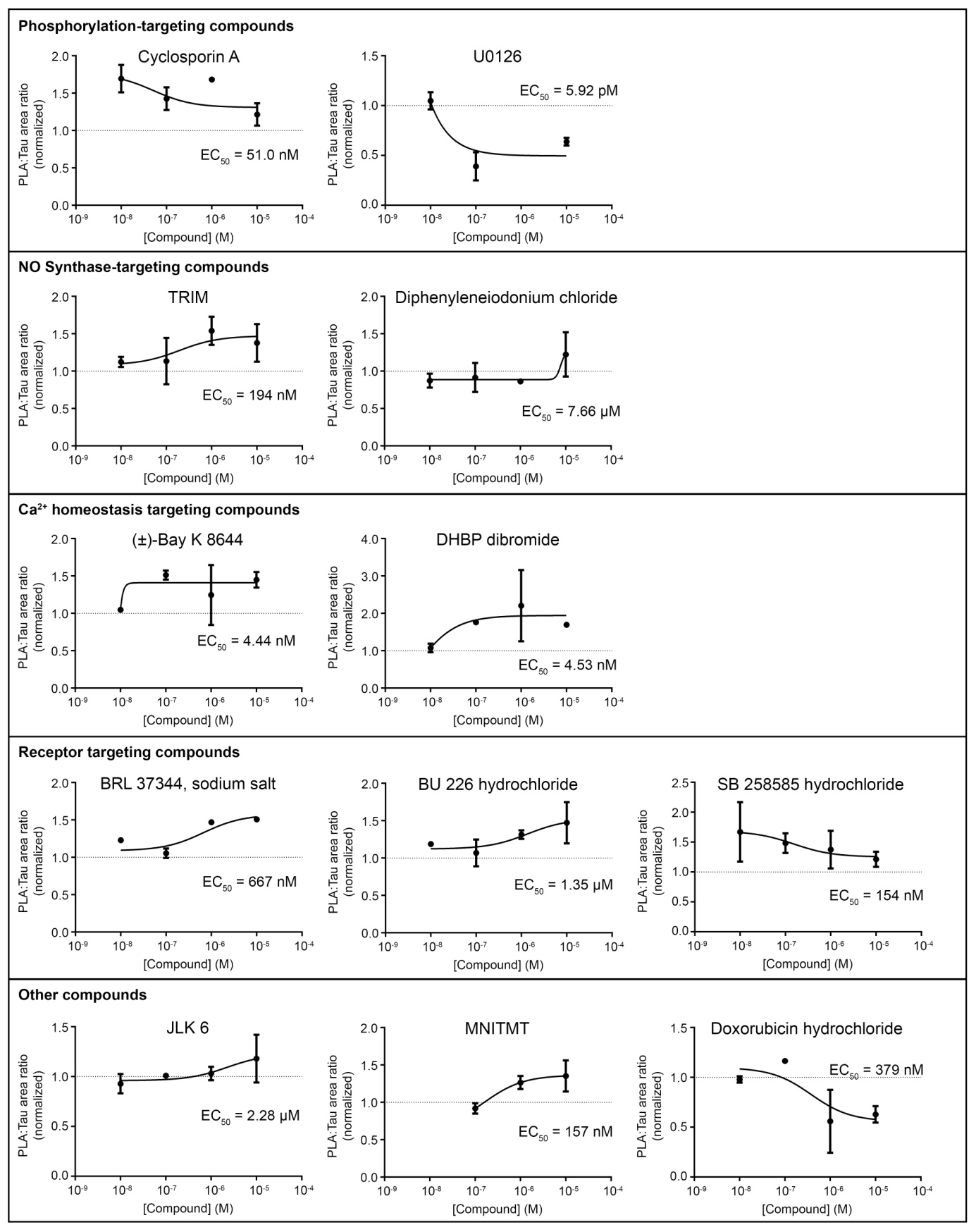
**

**Fig. S20** Dose-response curves and EC_50_ values for the 12 shortlisted compounds grouped according to their targets. See Fig. 4 for details. Phosphorylation targeting compounds: Cyclosporin A, an inhibitor of Calcineurin (through forming a blocking complex with Cyclophilin); U0126, a potent MEK inhibitor. NO-synthase targeting compounds: TRIM, a potent inhibitor of neuronal and inducible NO-synthases; Diphenyleneiodonium chloride, a GPR3 agonist that also inhibits NO-synthase and NADPH oxidase. Ca^2+^ homeostasis targeting compounds: (±)-Bay K 8644, a L-type Ca^2+^ channel activator; DHBP dibromide, an inhibitor of endoplasmic reticulum Ca^2+^ release. Receptor targeting compounds: BRL37344 sodium salt, a β3 agonist; BU 226 hydrochloride, a potent and highly selective I2 ligand; SB 258585 hydrochloride, a potent and selective 5-HT6 antagonist. Other compounds: JLK6, an inhibitor of γ-secretase-mediated βAPP processing; MNITMT, a non-toxic immunosuppressive agent; Doxorubicin hydrochloride, a tumor suppressor drug shown to inhibit DNA topoisomerase II and reduce Tau cellular levels.


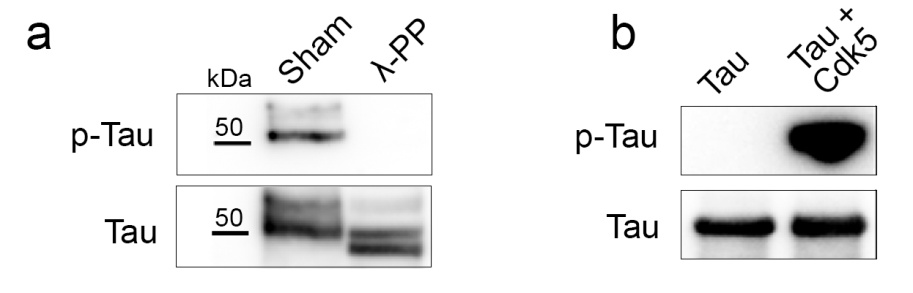


**Fig. S21** Modulation of Tau phosphorylation *in vitro*. **a.** Reduction of Tau phosphorylation at Thr 231 after 30 min incubation of PNC crude extracts with lambda protein phosphatase (λ-PP). **b.** *In vitro* phosphorylated recombinant Tau at Thr 231 is observed after incubation with recombinant Cdk5 for 1 h. Cdk2 dependent phosphorylation of Tau at Thr 231 has been published previously (Ref. 49).


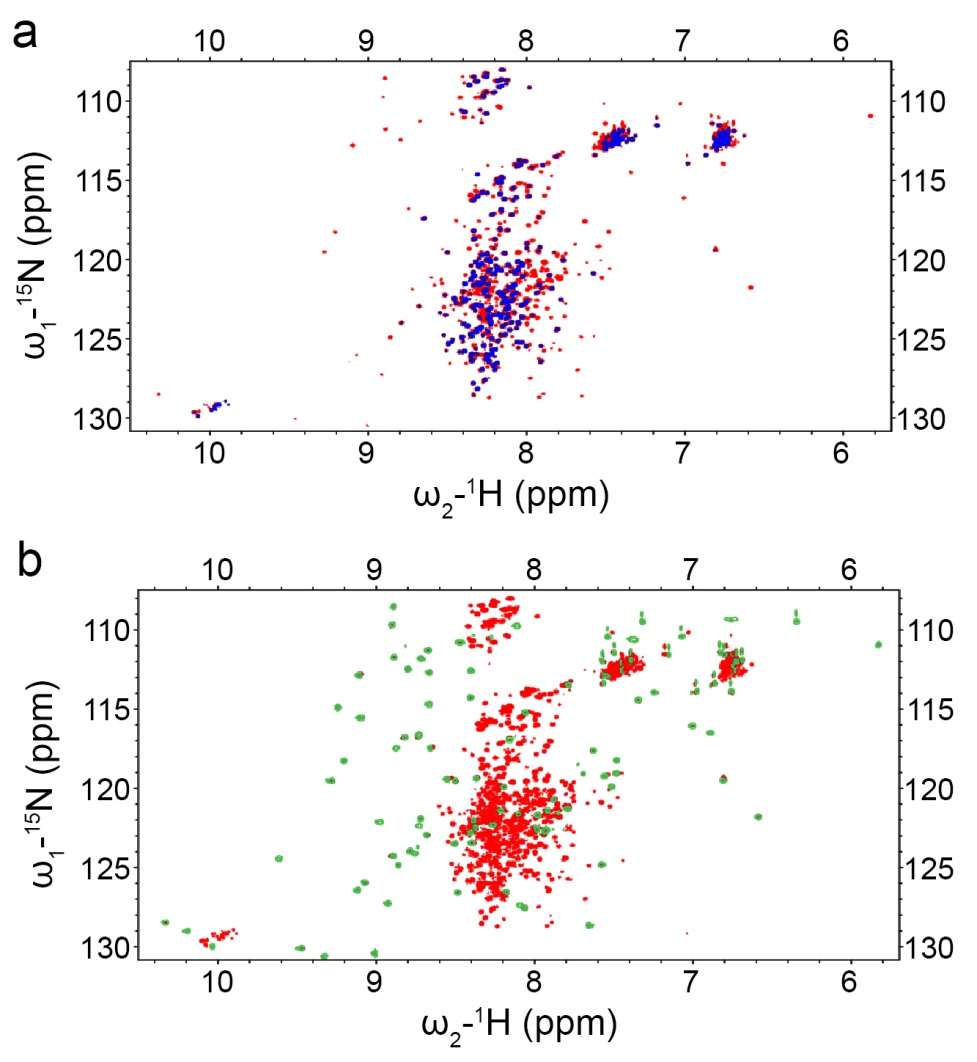


**Fig. S22** Additional signals in BIN1iso1-CLAP-T348E matched signals from BIN1 SH3 domain. Overlays of ^1^H-^15^N HSQC spectra of (**a**) BIN1iso1-CLAP-T348E protein (in red) and BIN1iso1 protein (superimposed in blue) or of (**b**) BIN1iso1-CLAP-T348E protein (in red) and BIN1 SH3 domain (superimposed in green). In BIN1iso1 spectrum, in blue, due to the large size of the BIN1iso1 protein, only signals corresponding to mobile disordered regions are detected. These signals show a typical poor dispersion on the ^1^H scale (ca. 7.5-8.5 ppm). Due to this intrinsic need of mobility to detect protein NMR signals, the SH3-BIN1 resonance are only detected when the domain behaves independently of the full protein, as is observed for BIN1iso1-CLAP-T348E (in red). Signals from the SH3-BIN1 domain show a good dispersion on the ^1^H scale (ca. 6.5-9.5 ppm), as expected for globular domain signals. The additional signals observed in BIN1iso1-CLAP-T348E, in red, matched signals from the SH3-BIN1 domain, in green.


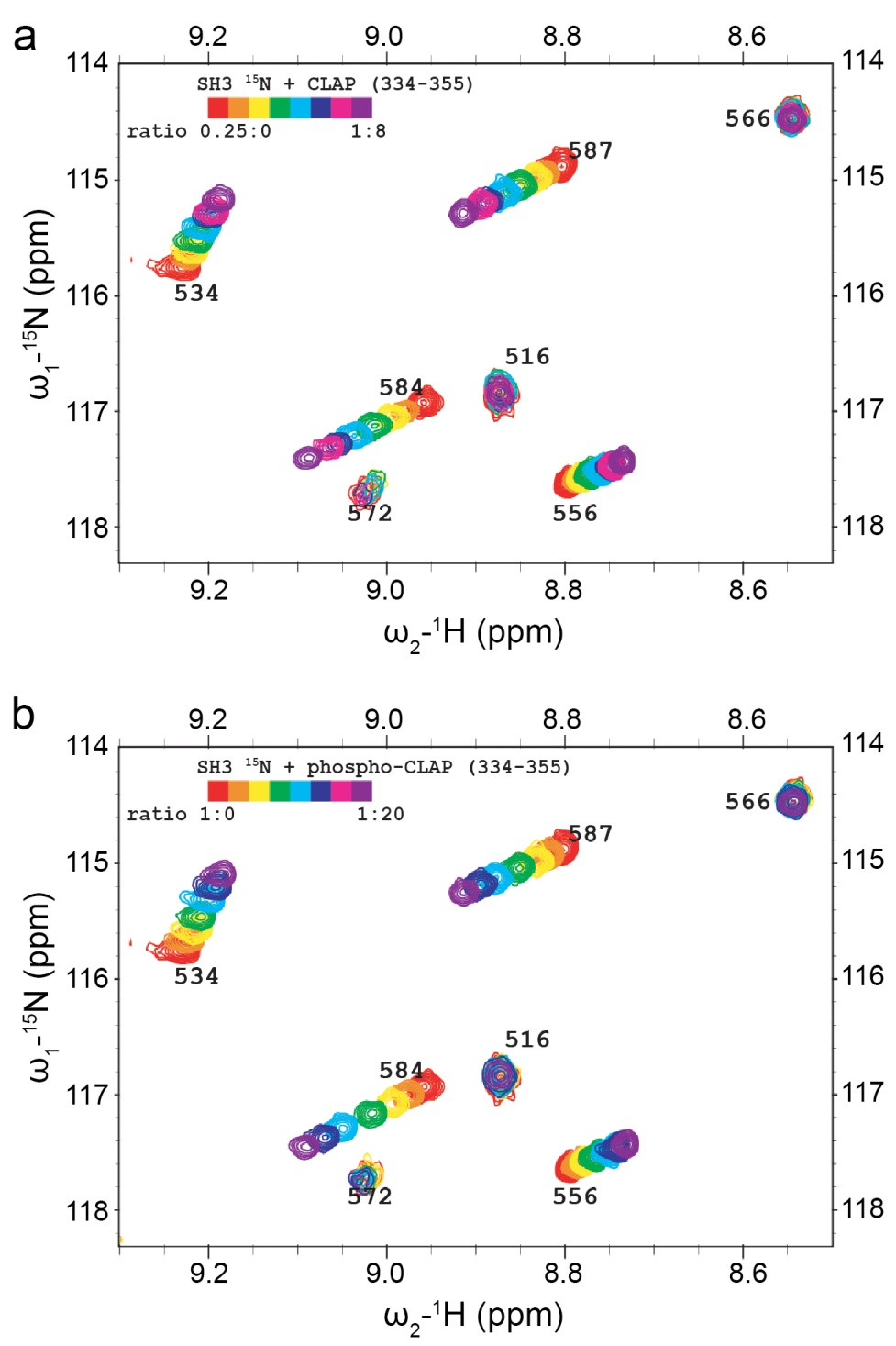


**Fig. S23** Titration of ^15^N-BIN1-SH3 with CLAP peptides. Detail of overlayed ^1^H-^15^N HSQC spectra of BIN1-SH3 domain, in the presence of increasing amount of (**a**) CLAP (334-355) peptide, molar ratios 0.25 to 8 (color scale, from red to violet), or of (**b**) phospho-CLAP (334-355) peptide, molar ratios 1 to 20 (color scale, from red to violet). One spectrum was recorded for each titration point. The gradual change of the chemical shift value for each resonance was then used to build a saturation curve (see Fig. 5g). Data were averaged to estimate the K_d_ values. Note that a larger excess of phospho-CLAP (334-355) peptide was needed to reach saturation, due to its lower affinity for BIN1-SH3 domain, compared to the non-phosphorylated peptide.

**
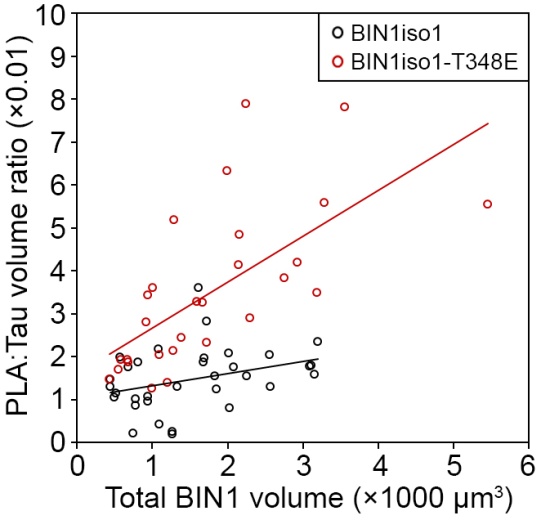
**

**Fig. S24** Correlation between BIN1-Tau PLA:Tau volume ratio and total BIN1 volume (based on BIN1 immunostaining) in BIN1iso1 and BIN1iso1-T348E. Each point represents one confocal image. Also see Fig. 5h-k.

**
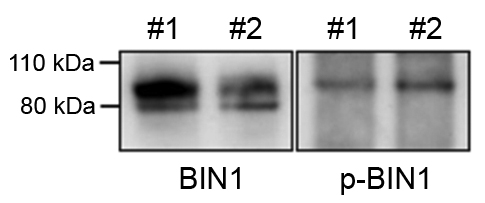
**

**Fig. S25** Immunoprecipitation using human brain samples. IP performed with total BIN1 antibody (99D) on human brain samples shows the specificity of p-T348 antibody towards phosphorylated BIN1.


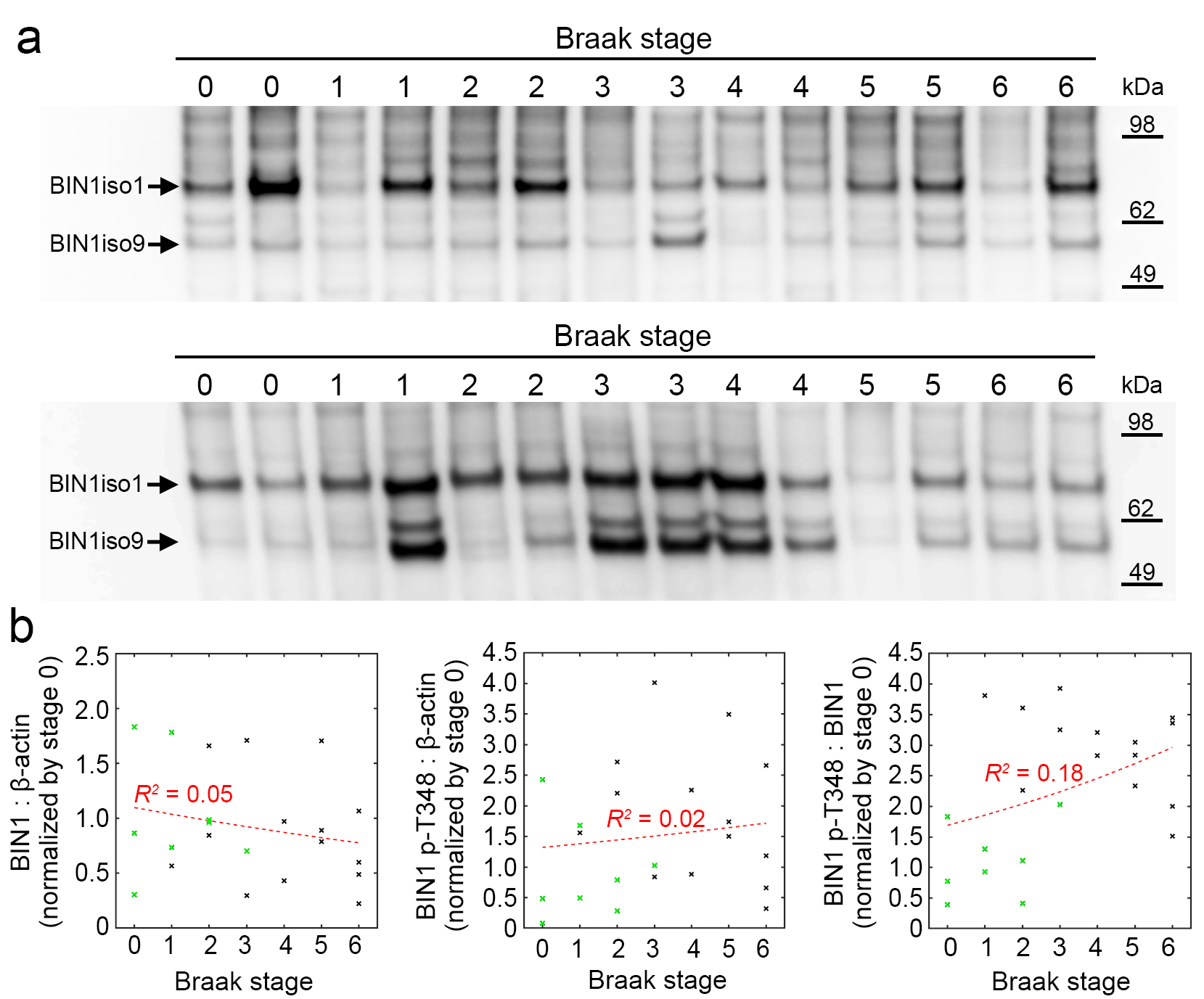


**Fig. S26** **a.** Uncropped immunoblots of human brain samples obtained with 99D antibody showing BIN1iso1 and BIN1iso9 bands. **b.** Quantification of the BIN1:β-actin, BIN1-p-T348:β-actin, and BIN1-p-T348:BIN1 signals, normalized with the mean of the control group (Braak stage = 0). Dashed red lines indicate exponential fits with coefficient of determination (R^2^). Residuals were normally distributed according to Anderson-Darling test with *p*-values 0.086, 0.174, and 0.580 for BIN1:β-actin, BIN1-p-T348:β-actin, and BIN1-p-T348:BIN1 signals, respectively. Data marked in green indicate non-AD cases according to neuropathological diagnosis.

**
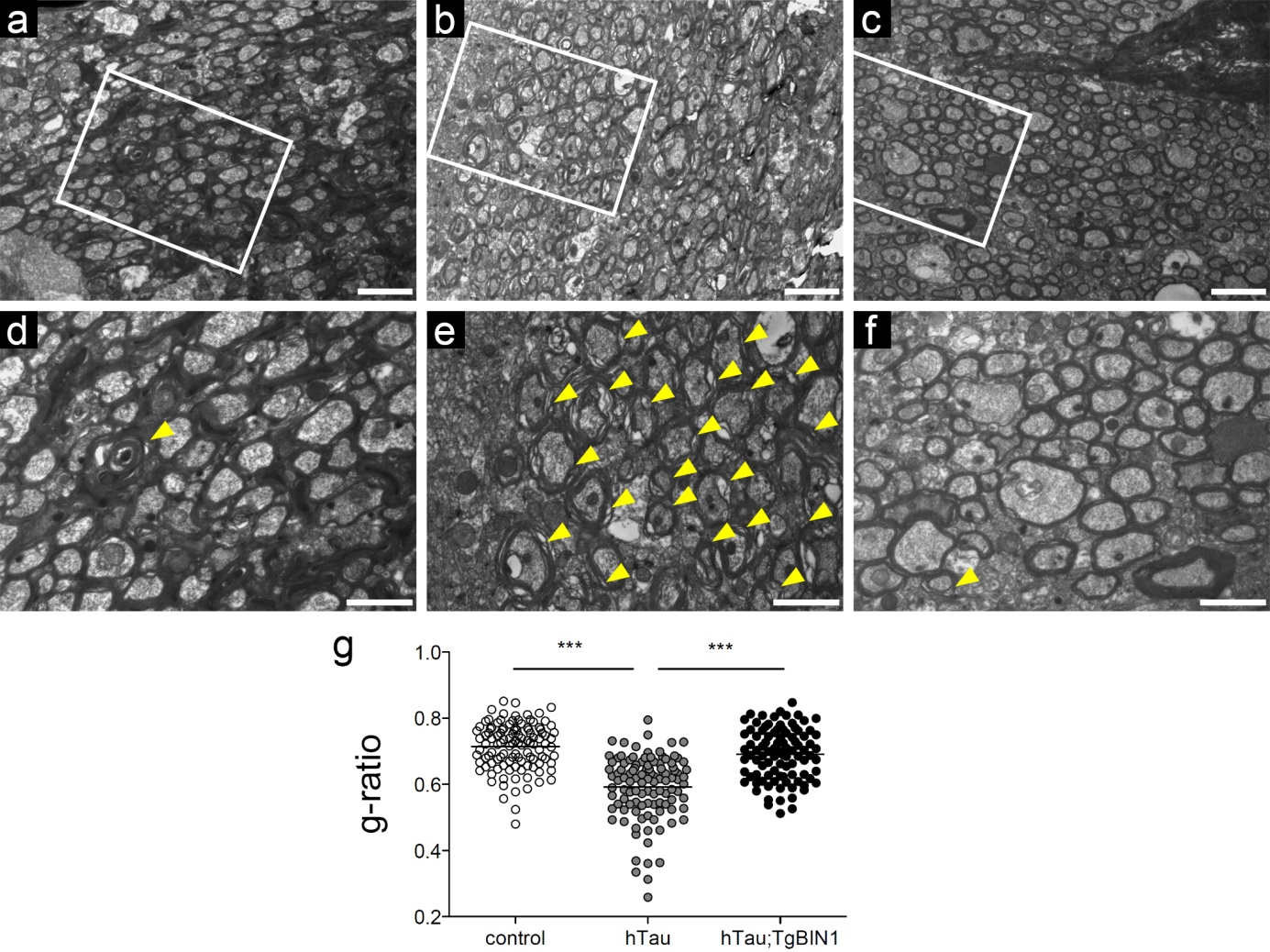
**

**Fig. S27** Myelin alterations following hTau overexpression was prevented upon BIN1 overexpression. **a-c.** Electron microscopy analysis of the myelinated axons in the fornices of 18-month-old control (A), hTau (B), and hTau;Tg*BIN1* (C) males. **d-f.** Serial magnification of the marked areas. Arrowheads point to myelin abnormalities defined by multiple myelin rings. Micrographs are representative of 2 animals per genotype. Scale bars = 5 µm (a-c) and 2 µm (d-f). **g.** G-ratio analysis of myelinated axon fibers in the fornices of 18-month-old mice. Dot plot based on 2 mice per condition and ca. 50 axons per mice. *** p < 0.001; ANOVA followed by Kruskal-Wallis post hoc test with Dunn’s multiple comparison.


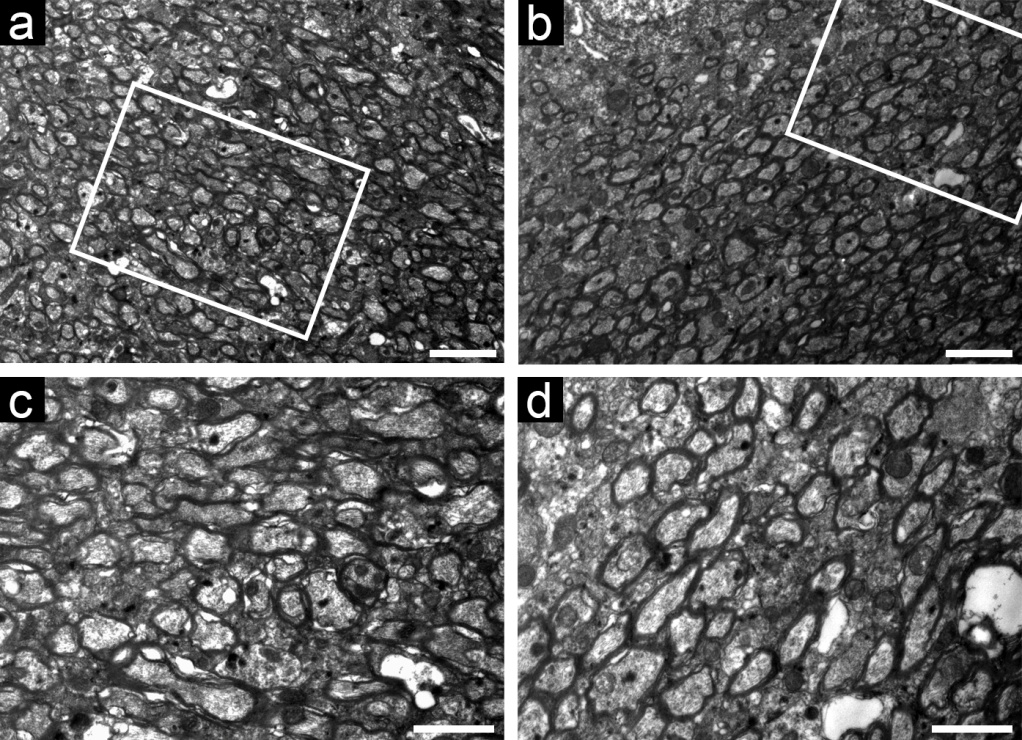


**Fig. S28** Myelin structure is unaffected following BIN1 overexpression. **a-b.** Electron microscopy analysis of the myelinated axons in the fornices of 18-month-old *Mapt^-/-^*;Tg*BIN1* (A) and *TgBIN1* (B) males. **c-d.** Serial magnification of the marked areas. Micrographs are representative of 2 animals per genotype. Scale bars = 5 µm (a-b) and 2 µm (c-d).

**
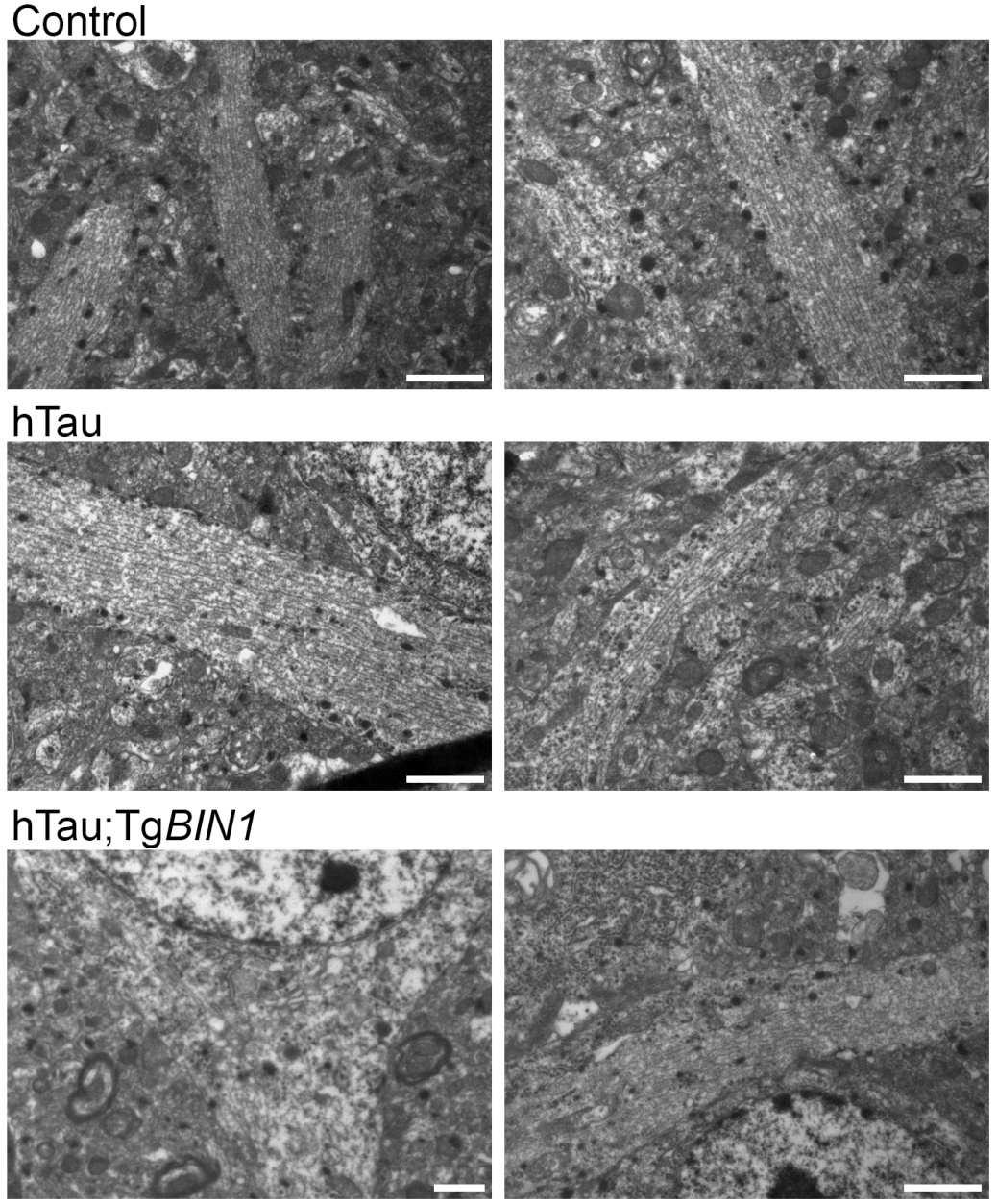
**

**Fig. S29** Axonal ultrastructure is unaffected in transgenic mice. Electron microscopy analysis of axon fibers in the hippocampal CA1 region of 18-month-old control, hTau, and hTau;Tg*BIN1* males. Micrographs are representative of 2 animals per genotype. Scale bars = 2 µm.
